# Early Pregnancy Targeted Exposome: Biological Response and Maternal BMI

**DOI:** 10.3390/toxics14050421

**Published:** 2026-05-12

**Authors:** Nadia Saadat, Soundara Viveka Thangaraj, Jasmin Chovatiya, Ravikumar Jagani, Syam S. Andra, Vasantha Padmanabhan

**Affiliations:** 1 Department of Pediatrics, University of Michigan, 7510 MSRB 1, 1500 W. Medical Center Drive, Ann Arbor, MI 48109, USA; svthan@umich.edu; 2 Institute for Exposomic Research, Department of Environmental Medicine, Icahn School of Medicine at Mount Sinai, New York, NY 10029, USA; jasmin.chovatiya@health.ny.gov (J.C.); ravi.jagani@mssm.edu (R.J.)

**Keywords:** exposome, pregnancy, maternal BMI, glucocorticoids, oxidative stress

## Abstract

Physiological and metabolic adaptations during pregnancy may increase susceptibility to harmful environmental chemicals. High maternal BMI that perturbs the metabolic, inflammatory, and hormonal milieus could modulate toxicant effects on pregnancy outcomes. First-trimester targeted urinary exposomes of 119 women (BMI ≥ 25 = 55; BMI < 25 = 64) from the Michigan Mother–Infant Pair cohort were profiled to assess how BMI influences urinary exposomes and related biological responses. A validated approach measured 96 chemicals and biomarkers of oxidative stress and glucocorticoids. Women in both groups reported similar lifestyles and consumer product use. Women with high BMI had lower urinary concentrations of several chemicals than women with low BMI. Phytoestrogens, polycyclic aromatic hydrocarbons, and insect repellants showed a higher magnitude of positive associations with other markers of exposure, oxidative and glucocorticoid stress in the high-BMI group, while UV filters, flame retardants, and phthalates showed a higher magnitude of positive associations with oxidative stress markers in the low-BMI group. The patterns of associations of exposure markers with stress markers and pregnancy outcomes (gestational age and birthweight) differed by maternal BMI. This highlights the importance of incorporating maternal BMI into the evaluation of exposure burden and its effects, as a factor that may actively shape biological responses.

## 1. Introduction

Rapid industrialization has significantly increased the production and use of synthetic chemicals, many of which can have adverse health effects. Lifestyle factors like smoking, the consumption of canned and highly processed foods, and personal product use can raise exposure to fine particulate matter, polycyclic aromatic hydrocarbons, volatile organic compounds, bisphenols, phthalates, etc. Social status also shapes exposure by influencing housing quality, neighborhood pollution, and job conditions, often concentrating multiple higher-exposure sources in lower-income groups [1]. Human biomonitoring studies confirm the presence of contaminants like bisphenols, pesticides, parabens, phthalates, per- and polyfluoroalkyl substances, polychlorinated biphenyls, dioxins, and flame retardants in human biological samples, illustrating the extent of human exposure [2,3]. Since real-world human exposure typically involves chronic, concurrent exposure to low doses of multiple chemicals throughout the lifetime, holistic approaches like the exposome framework are essential for accurate risk assessment [4] and enhancing disease prediction across the lifespan. While capturing all lifetime exposures remains challenging, advances in untargeted metabolomics [5] and targeted mass spectrometry-based multi-class assays [6] have enabled detecting up to 125 chemicals using a single biospecimen [7]. Despite these advances, comprehensive characterization of the urinary exposome remains limited, as few studies have systematically mapped multi-class chemical exposures using targeted urinary assays, even though urine provides a valuable non-invasive matrix for exposure assessment. We address this gap by employing targeted multi-class urinary assays in a pregnancy cohort, using a discovery-driven approach to identify environmental chemical factors that influence neonate health.

The first trimester of pregnancy represents a critical window of increased sensitivity to environmental exposures and heightened risk [8,9]. During this period of rapid fetal differentiation, maternal exposure to environmental chemicals can have lasting sex-specific effects on offspring health [10,11]. Both animal and human studies have demonstrated that exposures during this trimester are associated with sexually dimorphic impacts on cardiometabolic, respiratory, and neurodevelopmental outcomes in offspring [12,13,14], supporting Barker’s concept of developmental origins of health and disease [15]. Pregnancy is also a vulnerable stage for the mother, as physiological changes may amplify the effects of environmental exposures on her health [16]. Additionally, other maternal factors known to negatively affect maternal–fetal health may further modulate the impact of environmental chemical exposures.

In this context, maternal body mass index (BMI) has emerged as a significant determinant of both pregnancy outcomes and offspring health. Extensive evidence links higher maternal BMI to a variety of pregnancy complications, including increased risk of gestational diabetes mellitus, gestational hypertension, preeclampsia, venous thromboembolism, infection, and mental health problems [17,18,19]. For offspring, high maternal BMI is associated with an increased risk of respiratory tract infection [20], immune dysfunction [21], neurodevelopmental disorders [22], diabetes, hypertension, and cardiometabolic diseases [23,24]. Higher maternal BMI also increases the body’s chemical burden, particularly for lipophilic environmental chemicals, like persistent organic pollutants, bisphenols, and phthalates, that are sequestered in adipose tissues and may contribute to increased fetal exposure [25].

Obesity-related physiological changes can profoundly affect the storage, metabolism, and distribution of environmental chemicals. Increased adiposity enhances the accumulation of persistent organic pollutants, which disrupts adipose tissue function, elevates lipotoxicity, and triggers inflammation, thereby disrupting overall metabolism [26]. Additionally, obesity affects hepatic metabolism, renal handling, and tissue perfusion, modifying internal chemical doses, the formation of bioactive metabolites, and their delivery to target tissues [27,28,29]. Chronic low-grade inflammation, characteristic of obesity, further modifies immune function [30], which could potentially exacerbate the health impacts of toxic exposures. Obesity-associated physiological alterations also impact feto-placental cytochrome P4501A1 activity [31] and influence the kinetics, accumulation, and fetal transfer of lipid molecules [31,32,33], which are particularly relevant for lipophilic environmental chemicals. Conversely, several environmental chemicals function as obesogens (e.g., bisphenols and phthalates), exacerbating maternal weight gain [34,35,36] and establishing a feedback loop that can intensify the impacts of both obesity and chemical exposures. Despite these suggestive mechanistic pathways, current evidence lacks an in-depth mechanistic framework for how BMI modulates the relationship between chemical exposures and biological responses. Most studies treat BMI merely as a covariate or confounder, without directly investigating how obesity-induced changes in metabolism, endocrine function, and immune status influence the toxicokinetics and toxicodynamics of environmental chemicals, especially in pregnancy.

Given the complexity and ubiquity of concurrent exposures to multiple environmental chemicals, there is an urgent need for comprehensive, exposome-based investigations that evaluate cumulative and interactive effects, particularly during pregnancy, a period of heightened sensitivity and vulnerability for both mother and child. Despite prior pregnancy exposome studies demonstrating widespread, patterned chemical exposures among pregnant women [37,38,39], research remains scarce regarding how maternal physiological characteristics, such as BMI, may modulate both exposure profiles and biological responses. Overall, the focus of the study is innovative in applying targeted, multi-class urinary exposome profiling within a pregnancy cohort to systematically capture concurrent low-dose chemical exposures using a single biospecimen. It moves beyond traditional approaches by adopting a discovery-driven framework to link complex exposure patterns with maternal and neonatal health outcomes during a critical developmental window. Additionally, it uniquely positions maternal BMI not just as a confounder but as a potential biological modifier of exposure, integrating mechanistic perspectives into exposome research.

Our central hypothesis is that maternal BMI influences biological responses to chemical exposures, an area that remains largely overlooked in previous pregnancy exposome research. To address this gap, this data-driven study systematically characterizes the associations between multi-class urinary chemical exposure signatures and biological response markers (glucocorticoids and oxidative stress markers) in relation to maternal BMI during the first trimester of pregnancy and examines their potential implications on birth outcomes. Our approach aims to advance exposomics in maternal–child health and support evidence-based policy and clinical recommendations, establishing a foundation for future exposomic studies that consider individual susceptibility and physiologic modifiers alongside environmental exposures.

## 2. Materials and Methods

### 2.1. Study Population

The samples and demographic data used in this study were collected as part of the Michigan Mother Infant Pair (MMIP) cohort, a prospective pregnancy cohort approved by the Institutional Review Boards (IRB) at the University of Michigan (HUM00017941). Pregnant women were eligible to participate in the study if they (a) were 18–42 years of age; (b) had a singleton pregnancy; (c) were of any parity; (d) were not on fertility treatment; and e) delivered at the University of Michigan hospital. BMI in the study was calculated using participants’ body weight (in kilograms) divided by the square of their height (in meters). In this pilot study, urine samples and survey data from 119 participants were used. Participants were categorized as high-BMI (≥25 kg/m^2^; n = 55) and low-BMI (<25 kg/m^2^; n = 64) based on BMI calculated at the early pregnancy visit.

### 2.2. Recruitment and Sample Collection

Women were provided with the flyers at the registration of their first prenatal visit in the first trimester at Von Voigtlander Women’s Hospital. The research coordinator met with the women who showed interest in the study during normal office hours and answered questions regarding the study. Women who agreed to participate in the study completed the informed consent process and completed a survey that included maternal demographic characteristics, lifestyle factors (smoking, alcohol use, etc.), eating habits (canned foods, fresh fruits and vegetables, fast food, etc.), and use of consumer personal care products (perfumes, hair care products, and cosmetics) in the past three months. The pregnancy and birth data were abstracted from the medical records. Participants provided a urine specimen at the first-trimester prenatal visit (8–14 weeks of gestation) in polypropylene containers. The samples were aliquoted in glass vials and stored at −80 °C at the University of Michigan laboratory till subsequent analyses.

### 2.3. Targeted Multi-Class Urinary Biomarker Exposome Analysis

Urinary biomarker analyses on first-trimester urine specimens were undertaken at the Human Health Exposure Analysis Resource (HHEAR) Laboratory at the Icahn School of Medicine at Mount Sinai in New York, NY, USA. A validated multi-class analytical method was employed to quantify biomarkers of environmental chemical exposures and biological responses in human urine [6]. This multi-class approach facilitated the simultaneous analysis of 100 compounds across ten chemical categories, including organophosphate flame retardants (OPFRs), personal care and consumer product chemicals (PCPs), pesticides, phthalate and phthalate alternative metabolites (PHTH), phytoestrogens, polycyclic aromatic hydrocarbons (PAHs), tobacco smoke constituents, volatile organic compounds (VOCs), and biological response markers for oxidative and psychosocial stress. Complete analyte specifications, including CAS numbers, analyte codes, and chemical classifications, are provided in Supplementary Table S1.

The analytical workflow incorporated isotope-labeled internal standards and enzymatic deconjugation utilizing β-glucuronidase/arylsulfatase from *Helix pomatia*, along with solid-phase extraction (SPE) cleanup from 0.2 mL urine aliquots. Following extraction, chromatographic separation was achieved using Sciex’s Exion ultra-high-performance liquid chromatography (SCIEX, Framingham, MA, USA). This involved three sequential injections of the SPE extract on optimized LC columns: Hypersil Gold AQ for pesticides and psychosocial stress biomarkers, Betasil C18 for PCPs, PAHs, OPFRs, and VOCs, and Kinetex C8 for tobacco smoke and PHTH metabolites, phytoestrogens, and oxidative stress biomarkers. Detection and quantification were performed using a Sciex 6500+ triple quadrupole mass spectrometer (SCIEX, Framingham, MA, USA), which operated in multiple reaction monitoring mode with positive and negative electrospray ionization.

The method demonstrated exceptional sensitivity, with detection limits ranging from 0.04 to 2.0 ng/mL (Supplementary Methods, Table S4), precision characterized by relative standard deviations below 20%, and extraction recoveries between 80% and 110%. Batch-specific limits of detection (LOD) and quantification (LOQ) were calculated using replicate analyses of low-level spiked matrix blanks (n = 10) within the matrix to capture instrument noise and matrix-related variability. LOD was defined as 3× the standard deviation, and LOQ as 10× the standard deviation of these replicates. Quality assurance (QA) was upheld through participation in external proficiency testing (PT) programs, including the German External Quality Assessment Scheme [40,41] and the Canadian Organic Substances in Urine Quality Assessment Scheme (OSEQAS), conducted by the Centre de Toxicologie du Quebec (CTQ), and the HHEAR program’s quality control (QC) procedures [42]. Comprehensive analytical specifications, including sample preparation protocols, chromatographic parameters, and mass spectrometry conditions, are outlined in the Supplementary Methods.

To monitor and minimize batch effects and external contamination across the multi-class panel, each analytical batch included a comprehensive set of quality control samples and blanks that were processed and analyzed identically to study samples. Specifically, every run included: procedural (reagent) blanks, field blanks, matrix blanks (synthetic urine), and matrix-matched QC samples at low and high concentrations (unspiked urine QC pool; QC pool spiked at 1 ng/mL and 10 ng/mL of native analyte standards mixture) that were interspersed across the plate and injected with each set of unknowns (see Supplementary Methods Table S5 for characterized QC concentrations). Field blanks and procedural blanks were used to identify any analytes arising from sampling, transport, or laboratory reagents and were routinely inspected for detectable signals. Analytes observed in blanks were evaluated and, where appropriate, blank-corrected or reported as non-detected if signals could not be reliably distinguished from blank levels. Carryover and system cleanliness were further controlled by extensive autosampler rinses between injections and by using fresh SPE plates for each batch.

To assess and control batch-to-batch variability, we used isotope-dilution quantification with isotopically labeled internal standards added to every sample prior to enzymatic hydrolysis and extraction. Analyte signals were normalized to their corresponding internal standards, and calibration curves were prepared in matrix-matched standards over the analytical range with 1/x weighting to address heteroscedasticity. An automated liquid handling system (epMotion 5075vtc) was used to minimize handling variation across batches. Between-batch performance was tracked using the triplicate analyses of QC materials across five analytical batches (reported in Supplementary Methods Table S5), and acceptance criteria for precision and accuracy (RSD < 20% and recoveries within established ranges) were applied. The method’s performance was continuously evaluated with pooled urine QCs and inclusion of external proficiency samples (G-EQUAS and OSEQAS) stored from previous PT rounds to detect inter-batch shifts. Any observed batch bias was assessed and corrected using QC-based normalization. Any batch failing these criteria triggered an investigation and, if necessary, reanalysis. These procedures, together with documented limits of detection, recoveries, and precision in the Supplementary Methods, ensure that the multi-class assay sensitivity and recoveries reflect controlled, monitored performance across batches.

Urine creatinine concentrations were determined using a colorimetric methodology with a detection threshold of 0.3125 mg/dL, following the established protocol by [43]. The urine creatinine analysis is validated through G-EQUAS proficiency testing, allowing for creatinine normalization to correct for urine dilution. Urinary creatinine levels in high- and low-BMI groups are shown in Supplementary Figure S1.

### 2.4. Statistical Analysis

#### 2.4.1. Univariate Analysis

Targeted urinary exposome data were aligned to ng/dL concentrations for all chemicals and normalized using urinary creatinine levels (measured in mg/dL) as an internal standard (ng chemical/mg creatinine). The class-level data for each sub-chemical group represent the sum of normalized levels for all exposure chemicals measured in the specific class/subclass. The survey data were cleaned, and responses to questions (such as “yes” and “no”, etc.) were recoded to “1” and “2” before further statistical analysis using SPSS 29.0 (IBM corporation, Armonk, NY, USA).

#### 2.4.2. Bivariate Correlation Analysis

Bivariate Pearson Correlation analysis was used to investigate associations of class variables representing sub-chemical classes with the outcomes of interest (BMI) and lifestyle factors (survey responses). As the data was not normally distributed (Supplementary Figure S2), they were log-transformed prior to undertaking analyses for determining associations of chemical classes with maternal BMI and lifestyle factors (smoking, use of perfumes and cosmetics, haircare products, and consumption of canned food, fast food, and fresh vegetables, etc.). Additionally, bivariate correlation analyses with biological response markers (oxidative stress and glucocorticoid stress markers) and birth outcome measures (birthweight and gestational age at birth) and t-tests were performed on the log-transformed data to investigate differences in urinary levels of exposure classes (sub-chemical groups) and individual exposure markers (analytes) between low- and high-BMI groups (with Benjamini–Hochberg adjustment for false discovery rate) using SPSS v30, MetaboAnalyst v6, R version 4.5.2, and GraphPad prism 10.4.

#### 2.4.3. Multivariate Dimensionality Reduction Analysis

To investigate associations of exposure profiles (96 maternal urinary exposure signature) with maternal BMI, and biological response markers such as those associated with psychosocial stress/glucocorticoids (cortisol—CORTL and cortisone—CORTE) and oxidative stress (8-Iso Prostaglandin F2α (F2A8IP) and 4-Hydroxy-2-nonenal mercapturic acid (HNEMA)), dimensionality reduction modeling was applied and latent variables were calculated using SIMCA 18 (Sartorius Stedim Data Analytics, AB). Briefly, exposure variables normalized by urinary creatinine levels were unit-variance-scaled before unsupervised and supervised modeling. To get an overview of the data, unsupervised principal component analysis (PCA) was performed to observe trends and detect outliers. One outlier each from both low- and high-BMI groups, more than 3 standard deviations away, were removed based on principal component analysis (PCA, with the models with outliers shown in Supplementary Figure S3). Strong/major outliers have the tendency to pull the data towards them and affect the modeling of latent variables, calculating variance and visualization. For multivariate regression, Partial Least Squares (PLS) models were used. PLS uses weighted regression vectors that define the importance of each variable to create latent variables/composite variables for multiple responses [44], and Analysis of Variance of Cross Validated Residuals (CV-ANOVA) was used to assess model reliability, significance, and performance (Supplementary Methods, Tables S6–S9). This approach is suitable for exposome data with a large number of variables. The relationship between exposures and biological response variables in high- and low-BMI groups was explored; inner-relation plots and Variable Importance in Projection (VIP) were used to study patterns of associations and identify markers of these associations.

## 3. Results

### 3.1. Maternal Characteristics

Pregnant women in this study had a mean age of 31.68 ± 4.67. Seventy-three percent of the participants were non-Hispanic White, 17.6% were African American, 10 (8.4%) were Hispanic, and one participant reported as being more than one race. Nearly 54% had a BMI < 25, and 46% were overweight (BMI ≥ 25). More than half of the participants (54.6%) had an annual household income >75,000 USD, with 17.6% reporting a household income <25,000 (Table 1). Urinary creatinine levels were higher in the high-BMI group (*p* < 0.01) compared to the low-BMI group (Supplementary Figure S1).

### 3.2. Maternal Lifestyle Factors

In the self-reported questionnaires, women provided information about their lifestyle factors, eating habits, and use of consumer care products (Table 1). Six women reported being current or past smokers, and 11 women reported exposure to tobacco in the past 3 months. Only one woman reported living close to a landfill. In reference to the use of consumer products, the majority of the women reported the use of perfumes and cosmetics (79.8%) and haircare products (74.8%).

When asked about their dietary habits, more than half said yes to the use of canned foods (54.6%), with the majority of them consuming one or fewer servings per day. Barring two, all women reported consumption of fresh vegetables, with most consuming one to three servings per day. More than half of the participants (56.3%) did not consume fast food, with most consuming fast food once per week or less. In response to the question about the experience of stress, 48.7% said no and 44.5% said yes (Table 1). The frequencies of lifestyle factors in high- and low-BMI groups are detailed in the Supplementary Table S2, and no significant (chi-squared test) differences (in any of the variables) were observed between the two groups.

### 3.3. Association of Exposure Classes with Maternal Lifestyle

Few self-reported lifestyle factors were associated with exposure classes at the sub-chemical group level, although none remained statistically significant after FDR adjustment. In unadjusted analyses, history of smoking (current or past smoker) showed positive association with tobacco smoke metabolites (r = 0.20, *p* = 0.028), use of hair color showed positive association with fungicides (r =0.21, *p* = 0.023), and fast food consumption showed negative associations with fungicides (r = −0.21, *p* = 0.020) and VOCs (r = −0.19, *p* = 0.037) (Supplementary Table S3).

### 3.4. Exposure Classes and Maternal BMI

**Figure 1:**
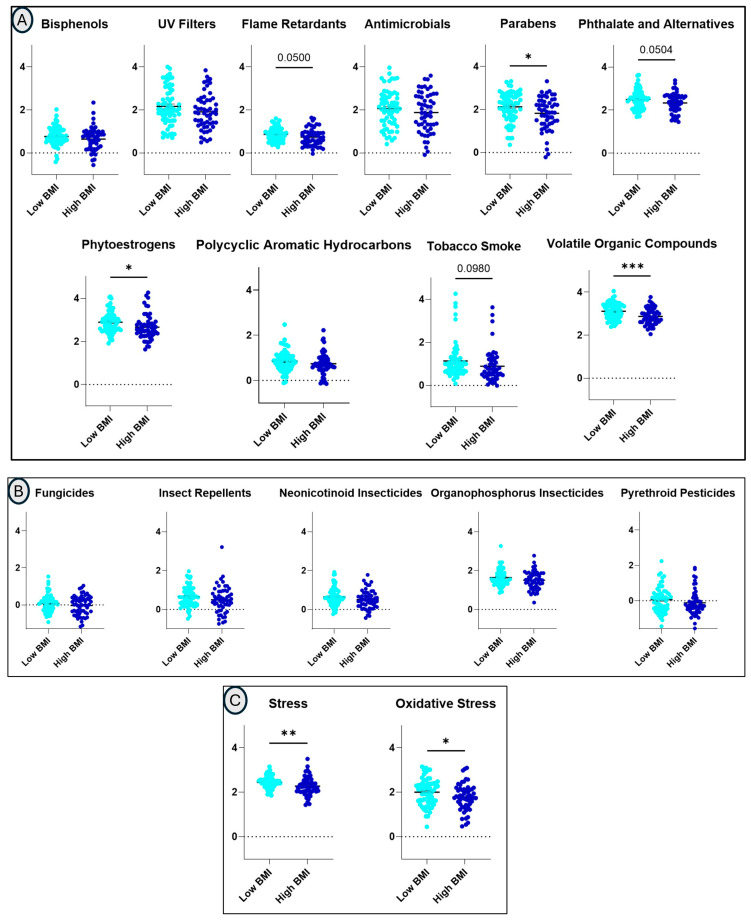
Differences in class-level urinary exposure profiles and biological response markers (glucocorticoid stress markers and oxidative stress markers) in low (<25)- and high (≥25)-BMI groups. Panels (**A**,**B**) show differences in exposure classes in low- and high-BMI groups. Differences in the glucocorticoid stress markers and oxidative stress markers in low- and high-BMI groups are shown in panel (**C**) (unadjusted *p* < 0.05 = *, *p* < 0.01= ** and *p* < 0.001= ***).

Differences in urinary levels of exposure classes in low- and high-BMI groups by t-test found significantly higher levels of VOCs in the low-BMI group (FDR-adjusted *p* = 0.011). In unadjusted analyses, urinary levels of VOCs (*p* = 0.001), phytoestrogens (*p* = 0.021), and parabens (*p* = 0.023) were lower in the high-BMI group (Figure 1 and Supplementary Table S4). Flame retardants (*p* = 0.050), phthalates and alternative metabolites (*p* = 0.050), and tobacco smoke metabolites (*p* = 0.098) also tended to be lower in the high-BMI group. Oxidative stress (*p* = 0.039) and psychosocial stress/glucocorticoid (*p* = 0.008) biomarkers were likewise lower in the high-BMI group (Figure 1 and Supplementary Table S4).

Investigation of maternal BMI as a continuous variable revealed inverse associations with parabens (FDR-adjusted *p* = 0.038) and VOCs (FDR-adjusted *p* = 0.015) that remained significant after FDR correction, while organophosphorus insecticides showed borderline significance (FDR-adjusted *p* = 0.05). In unadjusted analyses, BMI was negatively correlated with exposure chemical classes, including parabens (r = −0.26, *p* = 0.005), neonicotinoid insecticides (r = −0.19, *p* = 0.037), organophosphorus insecticides (r = −0.24, *p* = 0.01), pyrethroid pesticides (r = −0.19, *p* = 0.041), phthalate and alternative metabolites (r = −0.21, *p* = 0.022), phytoestrogens (r = −0.19, *p* = 0.041), and VOCs (r = −0.33, *p* < 0.001) (Supplementary Table S5).

### 3.5. Differences in Urinary Exposure Signatures Between BMI Groups

**Figure 2:**
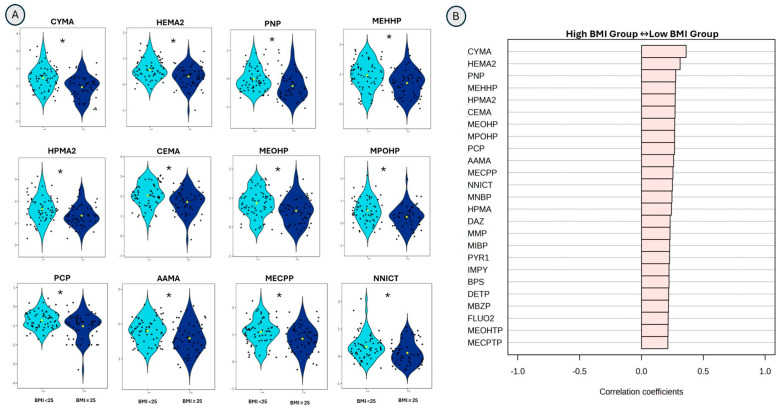
Differences in the individual exposure markers in low (<25)- and high (≥25)-BMI groups based on univariate analysis of the log-transformed targeted urinary exposome profiles. Panel (**A**) shows the top 12 exposure signatures that showed significant differences (*) based on the *t*-test with FDR-adjusted *p*-value < 0.05. Panel (**B**) shows the top 25 exposure signatures with correlation coefficients.

Twelve exposure signatures (individual analytes) were significantly lower in the high-BMI group compared to the low-BMI group after FDR correction (FDR-adjusted *p* < 0.05). These included VOC metabolites—N-Acetyl-S-(2-cyanoethyl)-L-cysteine (CYMA), N-Acetyl-S-(2-carboxyethyl)-L-cysteine (CEMA), N-Acetyl-S-(2-hydroxyethyl)-L-cysteine (HEMA2), N-Acetyl-S-(2-carbamoylethyl)-L-cysteine (AAMA), N-Acetyl-S-(2-hydroxypropyl)-L-cysteine (HPMA2)—insecticides and pesticides—4-Nitrophenol (PNP), pentachlorophenol (PCP), phthalate and phthalate alternative metabolites—Mono (2-ethyl-5-hydroxyhexyl) phthalate (MEHHP), Mono (2-ethyl-5-oxohexyl) phthalate (MEOHP), Mono-2-(propyl-6-oxoheptyl)-phthalate (MPOHP), Mono-(2-ethyl-5-carboxypentyl) phthalate (MECPP) and the tobacco metabolite nornicotine (NNICT). Correlation analyses were consistent with these results, showing inverse associations between BMI and these analytes, confirming that high BMI was consistently linked to reduced levels of these chemicals. Analytes significant at FDR-adjusted *p* < 0.05 and the top 25 analytes by correlation coefficients are shown in Figure 2A,B. Results of t-statistics, unadjusted *p*-values, and FDR-adjusted *p*-values are provided in Supplementary Table S6.

### 3.6. Relationship of Maternal BMI and Urinary Exposome Profiles with Biological Response Markers

Maternal BMI was negatively associated with glucocorticoid stress markers (*r* = −0.31, FDR-adjusted *p* = 0.001) and oxidative stress markers (r = −0.21, FDR-adjusted *p* = 0.026).

**Figure 3:**
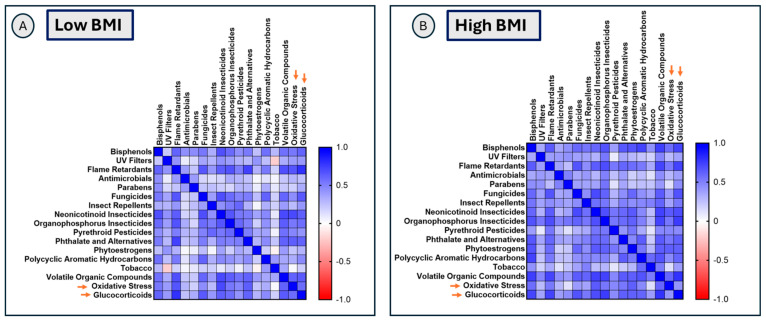
Differences in patterns of associations of exposure classes with biological response markers. Panel (**A**) shows the correlation matrix of exposure classes with oxidative stress and glucocorticoid stress markers (orange arrows) in the low-BMI group, with correlation coefficients and *p*-values listed in Supplementary Table S7. Panel (**B**) shows the correlation matrix of exposure classes with oxidative stress markers and glucocorticoid stress markers (orange arrows) in the high-BMI group, with correlation coefficients and *p*-values listed in Supplementary Table S7.

Overall, both BMI groups showed several significant (FDR-adjusted *p* < 0.05) positive correlations among exposure classes and between exposure classes, oxidative stress, and psychosocial stress/glucocorticoid stress markers. Exposure classes showed a higher magnitude of significant positive associations (FDR-adjusted *p* < 0.05) with other exposure classes in the high-BMI group compared to the low-BMI group (as reflected by darker shades of blue in Figure 3, and details are available in Supplementary Table S7). Patterns of associations between exposure classes and oxidative stress and glucocorticoid stress markers also differed by BMI group, with several exposure classes showing a higher magnitude of significant correlation (FDR-adjusted *p* < 0.05) with glucocorticoids in the high-BMI group. In particular, phytoestrogens, VOC, parabens, polycyclic aromatic hydrocarbons, and insecticides showed larger correlation coefficients in the high-BMI group compared to the low-BMI group, and these associations remained significant after FDR correction (Figure 3, Supplementary Table S7). UV filters, flame retardants, and phthalates showed a higher magnitude of positive associations with oxidative stress markers in the low-BMI group (FDR-adjusted *p* < 0.05) compared to the high-BMI group. Overall, maternal BMI appeared to differentially influence associations involving the glucocorticoid stress pathway. Notably, in the high-BMI group, phytoestrogens showed a higher magnitude of significant correlations with both oxidative stress (*r* = 0.63, *p* < 0.001) and psychosocial stress/glucocorticoid markers (*r* = 0.62, *p* < 0.001), which remained significant after FDR correction (Figure 3, Supplementary Table S7).

**Figure 4:**
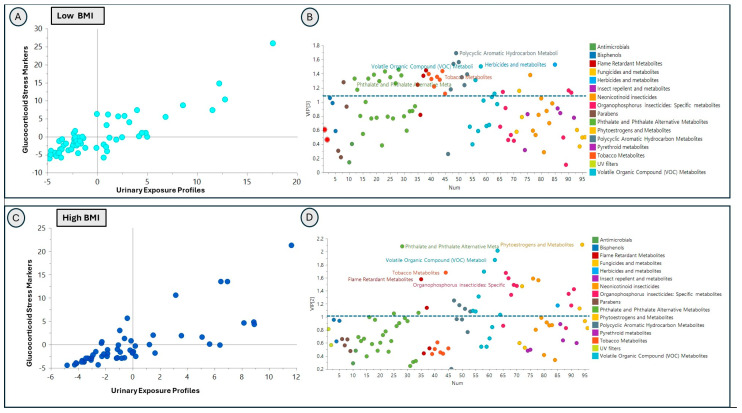
Association of exposome profile with glucocorticoid stress markers cortisol and cortisone in low-BMI (BMI < 25) and high-BMI (BMI ≥ 25) groups. PLS regression inner-relation plot showing the relationship of targeted urinary exposome profiles with glucocorticoid stress biomarkers in the low-BMI group (panel (**A**)); each score represents one participant). Panel (**B**) shows variables important in the projection (VIP) plot, showing the contribution of exposure classes to this relationship. Each point on the scatter plot represents one exposure marker colored by exposure class. PLS regression inner-relation plot showing the relationship of targeted urinary exposome profiles with glucocorticoid stress biomarkers cortisol and cortisone in the high-BMI group (panel (**C**)). Each score represents one participant. Panel (**D**) shows the VIP plot, showing the contribution of exposure classes to this relationship. Each point represents one exposure marker colored by exposure class.

**Figure 5:**
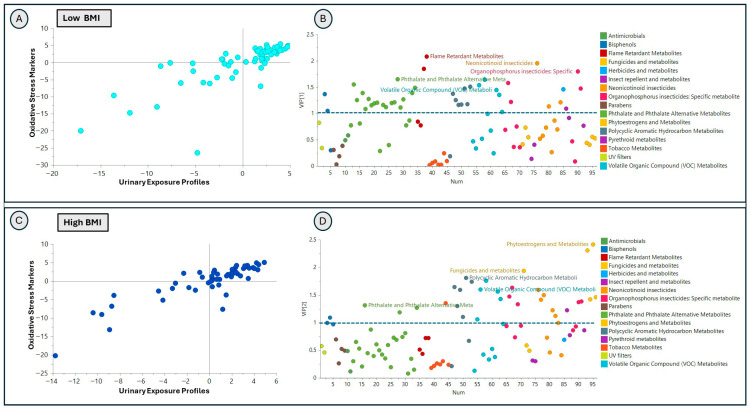
Association of exposome profile with oxidative stress markers (F2A8IP, HNEMA) in low-BMI (BMI <25) and high-BMI (BMI ≥ 25) groups. PLS regression inner-relation plot showing the relationship of targeted urinary exposome profiles with oxidative stress biomarkers in the low-BMI group (panel (**A**)), where each score represents one participant. Panel (**B**) shows the VIP plot, showing the contribution of exposure classes to this relationship. Each point represents one exposure marker colored by exposure class. PLS regression inner-relation plot showing the relationship of targeted urinary exposome profiles with oxidative stress biomarkers in the high-BMI group (panel (**C**)). Each score represents one participant. Panel (**D**) shows a VIP plot showing the contribution of exposure classes to this relationship. Each point represents one exposure marker colored by exposure class.

Dimensionality reduction supervised partial least square (PLS) regression models and identified associations of exposure profiles with glucocorticoid stress markers and oxidative stress markers in both the low-BMI (glucocorticoid stress R^2^ = 0.833 Q^2^ = 0.53, and oxidative stress R^2^ = 0.518, Q^2^ = 0.403) and the high-BMI (glucocorticoid stress R^2^ = 0.81 Q^2^ = 0.416, and oxidative stress R^2^ = 0.774 Q^2^ = 0.507) groups (Figure 4 and Figure 5). Cross-validated (CV) ANOVA was used to assess the overall model significance, and all models were statistically significant based on CV-ANOVA *p*-values (<0.05) (Supplementary Methods). The exposure signatures of individual analytes (represented by model VIPs on the y-axis) showed divergent association patterns with glucocorticoid stress markers between the low-BMI (Figure 4A,B) and high-BMI (Figure 4C,D) groups. Supplementary Table S8 lists the top 25 exposure analytes (chemicals) with the highest VIP values in models relating exposures to glucocorticoid stress biomarkers stratified by BMI groups. In the low-BMI group, the top VIP analytes indicative of higher-magnitude associations with significance included polycyclic aromatic hydrocarbons (2-Hydroxynaphthalene (NAP2), 2-Hydroxyfluorene (FLUO2), 1-Hydroxypyrene (PYR1)), herbicides (2,4-Dichlorophenoxyacetic acid (D24)), and VOCs (AAMA). In the high-BMI group, the top VIP analytes were phytoestrogens—Enterolactone (ETL), phthalate and phthalate alternatives—Mono-methyl phthalate (MMP), and VOCs—HEMA2, N-Acetyl-S-(3,4-dihydroxybutyl)-L-cysteine (DHBMA), and AAMA.

Similarly, urinary exposure profiles were also associated with oxidative stress markers in both the low- (Figure 5A,B) and high-BMI (Figure 5C,D) groups, with association patterns differing by BMI strata. The high-VIP analytes differed between the BMI groups and represented different chemical classes (Figure 5); the top 25 individual analytes with VIP values are listed in Supplementary Table S9. In the low-BMI group, the top five VIP analytes included flame retardants: Dibutyl phosphate (DPHP), Dibutyl phosphate (DBUP), neonicotinoid insecticides 6-Chloronicotinic acid (CINA6), organophosphorus insecticides 4-Nitrophenol (PNP), phthalates and phthalate alternatives (MMP). In the high-BMI group, the top five VIP analytes included phytoestrogens (Genistein (GNS) and Daidzein (DAZ)), fungicide (5-Hydroxythiabendazole (OHTBZ)), polycyclic aromatic hydrocarbon (2-Hydroxyphenanthrene (PHEN2)), and a VOC (AAMA).

Overall, at the individual exposure signature/analyte level, the majority showed positive associations with the biological response markers (two oxidative stress markers and two glucocorticoids) in both low- and high-BMI groups. However, the strength and significance of associations differed in the two BMI groups (Supplementary Table S10). Additionally, all stress markers showed significant associations with other stress markers (Supplementary Table S10).

### 3.7. Relationship of Maternal BMI and Urinary Exposome Profiles with Birth Outcome Measures

**Figure 6:**
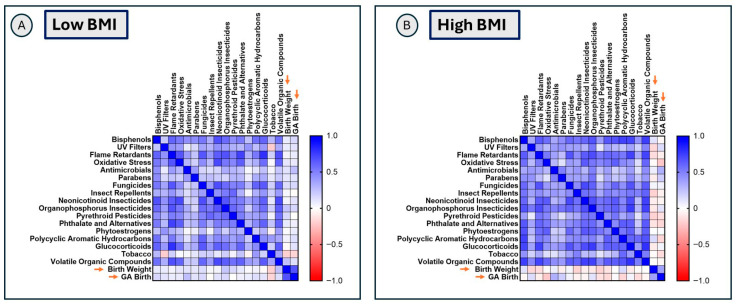
Association of exposome differences in patterns of associations of exposure classes with birth outcome measures. Panel (**A**) shows the correlation matrix of exposure classes with birthweight and gestational age (orange arrows) at birth in the low-BMI group. Panel (**B**) shows the correlation matrix of exposure classes with birthweight and gestational age at birth (orange arrows) in the high-BMI group.

Maternal BMI was not associated with gestational age, but it was weakly positively correlated with birthweight (r = 0.20 and *p* =0.037). The patterns of associations between exposure classes and birth outcomes (gestational age and birthweight) differed by BMI group. In the low-BMI group, most class-level correlations with gestational age and birthweight were positive but were not statistically significant (Figure 6A; Supplementary Table S11). In the high-BMI group, correlations were predominantly negative and likewise not statistically significant (Figure 6B; Supplementary Table S11). Antimicrobials were the only class positively associated (unadjusted) with gestational age in both low- (r = 0.25, *p* = 0.046) and high-BMI groups (r = 0.32, *p* = 0.021), with a larger correlation coefficient in the high-BMI group. Organophosphorus insecticides (r = 0.23, *p* = 0.074) and VOCs (r = 0.24, *p* = 0.056) showed a trend of positive association with birthweight (Figure 6 and Supplementary Table S11) only in low BMI group. However, none of the exposure–birth outcome associations remained significant after FDR adjustment.

In unadjusted analyses at the individual analyte level, none showed a significant association with gestational age in the low-BMI group, barring a negative trend of tobacco smoke metabolite—norcotinine (NCOTT, r = −0.23, *p* = 0.067) and bisphenol-4,4′-Cyclo-hexylidenebisphenol (BPZ, r = −0.22, *p* = 0.094)—and a positive trend of neonicotinoid insecticide-N-Desmethyl-acetamiprid (NDMA, r = 0.23, *p* = 0.071), as shown in Supplementary Table S12. In the high-BMI group, several phthalates and phthalate alternatives—Mono-2-ethyl-5-oxohexylterephthalate (MEOHTP), Mono-2-ethyl-5-hydroxyhexyl terephthalate (MEHHTP), Mono-2-ethyl-5- carboxypentyl terephthalate (MECPTP) and polycyclic aromatic hydrocarbon (PYR1)—showed negative associations, and phthalate Mono-(2-ethylhexyl) terephthalate (MEHTP) and antimicrobial compound Triclosan(TCS) showed positive associations (unadjusted *p* < 0.05) with gestational age. Phytoestrogen-Enterolactone (ETL, r = 0.26, *p* = 0.060) showed a trend of positive association with gestational age, as shown in Supplementary Table S12. In addition, oxidative stress marker 4-Hydroxy-2-nonenal mercapturic acid (HNEMA) showed a trend of negative association (r = −0.25, *p* = 0.076) with gestational age, but only in the high-BMI group.

The patterns of associations of the individual analytes with birthweight differed between the BMI groups in the unadjusted analysis. In the low-BMI group, several analytes, namely polycyclic aromatic hydrocarbon metabolite 3-hydroxyfluorene(FLUO3) (r = 0.31, *p* = 0.013) and pesticides dimethyldithiophosphate (DMDP) (r = 0.29, *p* = 0.021), dimethylthiophosphate (DMTP, r = 0.27, *p* = 0.035), and dimethylphosphate (DMP) (r = 0.25, *p* = 0.047) showed positive associations, while two of the tobacco smoke metabolites NCOTT (r = −0.282, *p* = 0.027) and 4-Hydroxy-4-(3-pyridyl)-butanoic acid (HYPYBUT) (r = −0.251, *p* = 0.049) showed negative association with birthweight (Supplementary Table S12). Additionally, pesticides trans-3-(2,2-dichlorovinyl)-2,2-dimethyl-cyclopropane-1-carboxylic acid (TDCCA) (r = 0.23, *p* = 0.068) and cis-3-(2,2-dichlorovinyl)-2,2-dimethyl-cyclopropane-1-carboxylic acid (CDCCA) (r = 0.23, *p* = 0.078), neonicotinoid insecticide clothianidin (CLO) (r = 0.23, *p* = 0.069), phthalate and phthalate alternative mono (3-carboxypropyl) phthalate (MCPP) (r = 0.22, *p* = 0.084), MECPP (r = 0.21, *p* = 0.096) and VOC (CEMA) (r = 0.22, *p* = 0.089) showed a trend of positive associations with birthweight (Supplementary Table S12). In the high-BMI group, analytes VOC HPMA2 (r = −0.31, *p* = 0.023), phthalate and phthalate alternative MEHHTP (r = −0.30, unadjusted *p* = 0.028), and pesticides/fungicides Cis-1,2,3,6-tetrahydrophthalimide (THPI, r = −0.29, unadjusted *p* = 0.036) showed negative associations, and neonicotinoid insecticide imidacloprid-olefin (OFIMI, (r = −0.26, unadjusted *p* = 0.063) and phthalate and phthalate alternative mono-2-ethyl-5- carboxypentyl terephthalate (MECPTP, r = −0.23, unadjusted *p* = 0.097) showed a trend of negative associations with birthweight (Supplementary Table S12). Signatures exhibiting a consistent inverse relationship with both gestational age and birthweight in the high-BMI group included the phthalate metabolite MEHHTP. For the low-BMI group, the tobacco metabolite NCOTT showed an inverse association with birthweight and a trend toward a negative association with gestational age at birth. Overall, the association of GA and birthweight with individual analytes failed to reach significance after FDR adjustment.

## 4. Discussion

Research on the impact of environmental chemicals and stressors during pregnancy, for the most part, has focused on linking exposures to pregnancy complications and adverse birth outcomes [45]. There is limited information available on how maternal metabolic and physiological state [16], such as higher BMI, may complicate interactions, toxicokinetics, bioaccumulation, metabolism, and the body’s response to multiple parallel environmental chemical exposures [25,26,46]. In most exposure studies, maternal BMI has been added to the statistical models or controlled as a factor, with very few studies investigating stratified analysis based on maternal BMI to understand its influence on exposure. Such data are crucial for informing effective prevention strategies aimed at improving maternal and child health. Using a targeted exposome approach to simultaneously assess 96 individual urinary chemical analytes and four biological response markers, findings from this study addressed the influence of low vs high BMI on the associations of urinary exposome with markers of biological response and birth outcomes. These findings revealed: (1) negative associations of urinary levels of several exposome chemicals with high maternal BMI and (2) the associations of chemical classes with other chemical classes, oxidative stress markers, glucocorticoid stress markers, and birth outcomes differed by maternal BMI status. In general, women with high BMI showed a higher magnitude of positive associations amongst exposure classes, including: (1) phytoestrogens, tobacco metabolites, insect repellents, and polyaromatic hydrocarbons with oxidative stress markers, and (2) phytoestrogens, tobacco metabolites, VOCs, parabens, polycyclic aromatic hydrocarbons, and organophosphorus insecticides with glucocorticoid stress markers. In contrast, the magnitude of associations of UV filters, flame retardants, and phthalates in women with high BMI was reduced. The implications of these findings for maternal and child health are discussed below.

### 4.1. Inverse Relationship of Maternal BMI with Urinary Exposome Profiles

BMI is a complex factor in exposome studies as it can serve as a confounder, mediator, effect modifier, or collider, depending on the specific context and health outcomes under investigation. The relationship between BMI and urinary concentrations of exposome chemicals is nuanced, with the directionality of associations varying by chemical, pharmacokinetics, study population, and data-handling approaches. However, most environmental exposure studies in pregnant women adjust for BMI as a confounding variable. Our study examined the influence of maternal BMI on the effects of environmental exposures and related biological response markers during pregnancy demonstrated a negative association between high BMI and urinary levels of several chemicals in early pregnancy. This finding contrasts with the mixed results reported in the literature, where associations between urinary chemical exposures and BMI often varied by analyte, age, sex, population, and study design [47,48]. Earlier NHANES data (2003–2006) reported a positive association between BPA and obesity in adults [49]. However, no significant association between BPA and maternal BMI was found in the present study, which is consistent with results from another study on a second-trimester pregnancy cohort [34]. Similarly, phthalate metabolite associations with BMI were found to be influenced by age and sex, with positive association of urinary MEHHP and DEHP metabolites with BMI in older women [50] and no association in pregnant women [34]. However, we observed lower urinary concentrations of phthalate metabolites such as MEHHP, MEOHP, MPOHP, and MECPP in individuals with high BMI, underscoring an inverse relationship in our cohort. Regarding tobacco exposure, our study found a negative association between maternal BMI and the tobacco metabolite nornicotine, in contrast to another study that showed no association between maternal BMI and another metabolite of tobacco, cotinine [51]. Studies in other populations have found urinary nicotine and 2-naphthol levels to be positively associated with BMI [52,53], highlighting the complexity in exposure biomarkers in relation to BMI across different exposure metrics and study populations.

The observed inverse relationship between BMI and urinary concentrations, particularly for lipophilic or bioaccumulative toxicants, may be attributable to several factors rather than low exposure. Greater adiposity provides a larger storage compartment for lipophilic chemicals, reducing the fraction available for renal elimination [25], influences distribution and clearance rates, and alters diet and behaviors affecting exposure, thereby affecting metabolite profiles in urine. Furthermore, obesity is linked to glomerular hyperfiltration [54,55] and can modify tubular handling, liver metabolism, biliary excretion, and overall pharmacokinetics, all of which may alter the rate and form in which chemicals are excreted in urine [56,57,58]. Pregnancy produces overlapping physiologic changes like expanded plasma volume [59], glomerular hyperfiltration [60,61,62], changes in hepatic nutrient metabolism [63], and drug pharmacokinetics [64] that can further shift urinary kinetics. Consequently, in individuals who are pregnant and/or have higher BMI, lower urinary concentrations do not necessarily indicate lower total exposure or body burden. Since the questionnaire was not detailed enough to address significant lifestyle differences, key determinants of exposure intensity (e.g., product brands/ingredients, packaging contact, cooking practices, and detailed diet composition) cannot be captured. In addition, limited power and measurement error could also mask group contrasts. Therefore, the observed BMI–urinary concentration relationship likely reflects a combination of exposure-source variability and physiologic differences affecting uptake, metabolism, and excretion.

### 4.2. Influence of Maternal BMI on Stress Markers

The complexity posed by dysregulated physiological and metabolic pathways with maternal obesity is reflected in the response to exposures and stressors. Glucocorticoid stress [65,66] and oxidative stress [67] pathways have been shown to be dysregulated in pregnant women with high BMI. Consistent with our findings, other studies have reported lower levels of glucocorticoids in women with high BMI [65,66,68,69]. Similarly, urinary cortisol was found to be lower in women with high BMI, despite reporting higher perceived stress, indicative of an altered response to psychosocial stress [65]. Maternal obesity has been linked to higher circulating oxidative stress markers in pregnant women and their babies [67], with increased risk of pregnancy complications, including preeclampsia, gestational diabetes, and adverse birth outcomes [67]. The lower levels of urinary oxidative stress markers in women with high BMI found in the present study may not be reflective of circulating oxidative stress markers, such as those reported in other studies, potentially due to differences in clearance mechanisms. Physiological changes and adaptations during pregnancy in women with high BMI may play a role in the modified response to exposures and stressors, potentially influencing birth outcomes, including birthweight [66,70], as has been observed in our study. We used the innovative multi-class assay to quantify 96 chemicals and stress markers concurrently in the same aliquot from each participant, enabling internally comparable correlation analyses across analytes under identical pre-analytical conditions. To avoid any differences in processing, the low- and high-BMI samples were processed similarly, stored at −80 °C, and thawed once for aliquoting before the exposome analysis. Any differences may therefore reflect factors related to obesity-associated physiology (e.g., altered renal handling/creatinine clearance and hyperfiltration). Since the multi-class panel provided limited information about the overall oxidative stress status, further investigation involving a panel of stable oxidative stress markers needs to be considered in future studies encompassing both blood and urinary markers.

### 4.3. Differences in Patterns of Associations of Exposome Profiles with Biological Stress Response Markers

Positive associations of exposure classes and individual analytes with oxidative stress and glucocorticoid stress markers in both low- and high-BMI groups may indicate higher stress response to environmental exposures, independent of maternal BMI. For instance, AAMA, a urinary metabolite (a mercapturic acid) [71,72] of the volatile organic compound acrylamide (present in heated/fried starchy food, etc.), a known stable marker for acrylamide exposure, was identified as a factor related to glucocorticoids and oxidative stress levels in pregnant women from both high- and low-BMI groups in our cohort. Higher acrylamide exposure was shown to affect fetal growth and was linked to low birthweight and babies who were small for gestational age [73,74,75]. Our results of higher oxidative stress and glucocorticoid levels in response to maternal environmental chemical exposure are consistent with other reports in the literature [76,77,78,79]. Uncontrolled higher levels of oxidative stress and higher glucocorticoid levels during pregnancy may lead to pregnancy complications and adverse pregnancy outcomes [76,77,78].

Interestingly, the patterns of associations of the exposome classes with glucocorticoids and oxidative stress markers differed by BMI group, with different chemical classes driving the positive associations in women with low versus high BMI. Differential influence of BMI on the impact of exposures on stress pathways may be related to the chemical nature of chemical mixtures, their lipophilicity, and/or possible accumulation in body tissues or elimination.

Several classes of chemicals, which showed a higher magnitude of positive associations with stress markers in women with high BMI in our study, have been shown to contribute to pregnancy complications and adverse birth outcomes. These include phytoestrogens [80,81,82,83], VOCs [84], parabens [85], polycyclic aromatic hydrocarbons [86], tobacco metabolites, insect repellents, and insecticides [87]. A higher magnitude of associations between exposure classes and glucocorticoid stress markers in women with high BMI may indicate a possible higher susceptibility of the high-BMI group to the exposure effects of these chemicals. Furthermore, a higher magnitude of positive associations of chemical classes with other chemical classes in the high-BMI group may affect the potential for additive and/or synergistic effects of combined exposures. Although it cannot be confirmed in this study, these findings may indicate the possibility of bioaccumulation of toxicants in women with high BMI [78], contributing to a higher magnitude of positive associations.

The strength of positive associations of phytoestrogens with both oxidative stress and corticosteroid stress markers in women with high BMI is suggestive of a greater impact of phytoestrogen exposure on both stress pathways. Phytoestrogens are associated with endocrine disruption and are reported to have mixed effects on pregnancy, affecting pregnancy outcomes and the baby’s development [82,88]. Human studies are not as conclusive as animal studies in explaining the effects of phytoestrogens on stress pathways and pregnancy outcomes [83,89]. Preclinical studies on adrenal cortical cells indicate phytoestrogens reduce cortisol levels [90]. Clinical studies have been mixed, with many studies showing no effects on glucocorticoids [91,92].

The finding of a higher magnitude of associations of UV filters, flame retardants, and phthalate metabolites in the low-BMI group compared to the high-BMI group needs further investigation. UV filters, although designed to reduce reactive oxygen species damage, were found to increase oxidative stress, alone and in co-exposures with other chemicals, including phthalates [93]. To avoid complications and harm to the developing baby, the use of physical barriers and sunscreens without harmful chemicals is recommended during pregnancy [94]. Similarly, organophosphate flame retardants are linked to higher oxidative stress [95]. However, the associations of these chemicals with oxidative stress and low BMI during pregnancy are not well characterized in the literature.

### 4.4. Maternal BMI, Exposome Profiles, and Birth Outcomes

Maternal pre-pregnancy high BMI has been linked to adverse birth outcomes such as macrosomia and lower gestational age at birth [96]. High BMI is also a risk factor for gestational diabetes and preeclampsia, further contributing to the risk of pregnancy complications and adverse pregnancy outcomes [97].

Because this was a small pilot study, it was underpowered to detect associations with birth outcomes, for which effect sizes are typically modest. The trend of negative associations of gestational age and birthweight with several phthalates and phthalate metabolites in women with high BMI suggests the potential of higher susceptibility of these women to the adverse impact of phthalates. Specifically, MEHHTP, a breakdown product of DEHP, showed negative associations with both gestational age and birthweight in women with high BMI. Phthalates are considered obesogens and tend to facilitate fat accumulation [98]. Furthermore, their lipophilic nature increases the likelihood of bioaccumulation in adipose tissue during pregnancy, contributing to metabolic and endocrine perturbations and adverse birth outcomes [99], as well as to sustained post-pregnancy weight gain [100].

The trend toward a negative association of the tobacco metabolite norcotinine with birthweight and gestational age in women with low BMI is consistent with an earlier report on the effects of tobacco smoke on birthweight [101]. Supportive of this, a study from Japan also reported that the association of maternal smoking with gestational weight gain and low birthweight may be modified by maternal BMI [102].

### 4.5. Implications and Risk Assessment

Our findings highlight the need to consider maternal BMI in risk assessment measures and to establish guidelines for mitigating the negative impact of environmental exposures in pregnant women. Research has consistently shown the negative impact of environmental exposures on oxidative and psychosocial stress, leading to adverse pregnancy outcomes and the developmental programming of the offspring [76,77,78]. A higher magnitude of positive associations of urinary exposome with stress markers in the high-BMI group might indicate more susceptibility to the adverse effects of exposures to environmental chemicals on stress pathways. Similarly, the differences in patterns of associations of urinary exposure markers with gestational age and birthweight in low- and high-BMI groups point toward the differential, BMI-dependent effects of the chemical exposures on pregnancy outcomes. More appropriate strategies should be investigated to mitigate the adverse impact of exposures on pregnancy in women.

### 4.6. Strengths and Limitations

The major strength of the study is the simultaneous assessment of targeted urinary exposome profiles with 96 analytes and four stress markers, which enabled us to investigate the impact of maternal environmental exposures in relation to maternal BMI on biological response markers (oxidative stress and glucocorticoids) and pregnancy outcomes (gestational age and birthweight). To address the high correlation structure typical of multi-chemical biomonitoring data, we employed a dimensionality reduction PLS approach. PLS is well-suited for correlated variables and estimates latent variables to assess associations with stress markers. Hence, we retained all 96 analytes rather than filtering based on multicollinearity to understand the relationship of exposure signatures with stress markers. Another strength of the study is the assessment of exposure profiles during early pregnancy (first trimester), a critical sexually dimorphic window of fetal development. However, there are several limitations to the findings of this study. The findings from this study point to patterns of associations of exposures with stressors at the same time point, as no such causal inference can be made based on these findings. Furthermore, the temporality of the bidirectional nature of the exposure and stress association cannot be investigated using the cross-sectional study design used in this pilot study. As this is a pilot study with a smaller sample, recommended measures were applied to reduce the bias. These include: (1) both high- and low-BMI groups being recruited in the same setting/cohort; (2) all the races being represented in both the low- and high-BMI groups; and (3) confirming by a chi-squared test that the two groups did not differ in demographic and lifestyle factors. Second, the interpretation of the findings is based on the assumption that the exposure risks were similar between the low- and high-BMI groups. These assumptions are based on the following facts: (1) the study participants were recruited from prenatal clinics at the suburban prenatal clinic/hospital in Michigan, and (2) no significant differences were observed in the lifestyle factors between the BMI groups. The third limitation is that the urinary exposure assessment is limited to the metabolites excreted in the urine and may not provide a full picture of the overall internal burden of the exposures in biofluids and bioaccumulation in the tissues. As several exposure signatures are recommended to be measured in urine and are not reliably detected in biofluids, a urinary targeted exposome investigation was performed. Urinary creatinine and associated exposure signature levels can be affected by metabolic changes with higher maternal BMI, hyperfiltration, and bioaccumulation of these chemicals. In addition, targeted exposome analysis only included 96 chemicals, and, although representative, it is not a complete analysis, and only a subset of the chemicals to which humans are exposed was measured. Survey data on lifestyle factors and consumer/personal care product use were limited; therefore, follow-up studies should incorporate more detailed measures (e.g., frequency, duration, and product types/brands) to better characterize these exposures and clarify their potential role. We could not assess total water consumption as the data was collected at routine prenatal visits for this retrospective observational study. In future studies, total water intake should be assessed and considered in analyses of maternal BMI; alternatively, standardized first-morning urine samples, which better reflect longer-term fluid balance, could be used to reduce variability due to hydration status [103]. Although exposures and stress markers were measured in early pregnancy and are biologically plausibly linked, our data were collected at a single time point and therefore cannot determine whether exposures contribute to stress responses or whether the underlying stress state and related physiological changes alter chemical metabolism and urinary excretion. Accordingly, these findings should be interpreted as concurrent associations rather than causal effects, and longitudinal studies with repeated exposure and stress measurements are needed to evaluate temporality and bidirectionality. Finally, caution should be exercised in generalizing these findings to pregnant or non-pregnant women in other environmental settings, as the cumulative burden of exposures may vary. Similarly, the findings from this study cannot be generalized to other populations due to the homogenous demographic and socioeconomic characteristics in our sample, but may provide help in establishing a framework for similar investigations. Since people are exposed to complex mixtures, future research should quantify cumulative exposure as synergistic or additive effects among chemicals may shape both urinary excretion profiles and potential health outcomes.

## 5. Conclusions

The findings from this exploratory study highlight the complex relationship of maternal body mass index with urinary exposome that potentially affects biologic response markers and birth outcomes measures, namely, gestational age and birthweight. The inverse associations of urinary exposure markers with high BMI during pregnancy could be related to complex metabolic and physiological changes related to high BMI. These changes may include changes in toxicokinetics, bioaccumulation, and hyperfiltration of environmental chemicals. Our cross-sectional observational study found differences in patterns of associations of exposures with oxidative and glucocorticoid stress markers and birth outcomes between low- and high-BMI groups. Furthermore, a higher magnitude of positive associations of several exposure classes and individual analytes with stress markers may indicate differences in susceptibility to the impact of these environmental chemicals in women with high BMI compared to those with low BMI. Although these findings are of interest, they do not permit causal conclusions and should be followed up with longitudinal studies in larger cohorts.

## Figures and Tables

**Table 1:** Maternal characteristics and lifestyle factors.

		N	%
Age	31.68 ± 4.67	119	
Race			
Black/African American		21	17.6%
Hispanic		10	8.4%
More Than One Race		1	0.8%
Non-Hispanic White		87	73.1%
BMI			
BMI < 25		64	53.80%
BMI ≥ 25		55	46.20%
Household Income			
<25,000		21	17.60%
25,000–49,000		12	10.10%
50,000–74,999		21	17.60%
>75,000		19	54.60%
Smoker	No	112	94.10%
	Yes	6	5.00%
	Missing	1	0.80%
Smoke/day	0/Day	115	96.60%
	1–3/Day	1	0.80%
	1/day	1	0.80%
	3/day	1	0.80%
	1 every 3 days	1	0.80%
Tobacco Exposure in Last 3 Months	No	108	90.80%
	Yes	11	9.20%
Living Near Landfill	No	115	96.60%
	Not sure	1	0.80%
	I don’t think so	1	0.80%
	Potentially	1	0.80%
	Yes	1	0.80%
Use of Perfumes and Cosmetics	No	22	18.50%
	Yes	95	79.80%
	Missing	2	1.70%
Haircare Products	No	28	23.50%
	Yes	89	74.80%
	Missing	2	1.70%
Dental Fillings in Last 3 Months	No	111	93.30%
	Yes	8	6.70%
Can Foods Consumption	No	53	44.50%
	Yes	65	54.60%
	Missing	1	0.80%
Can Food Frequency	1 serving or less/day	61	51.30%
	1 serving or less/week	1	0.80%
	2–3 servings/day	4	3.40%
	No canned food	53	44.50%
Fast Food Consumption	No	67	56.30%
	Yes	51	42.90%
	Missing	1	0.80%
Fast Food Frequency	<1/week	1	0.80%
	1/week	39	32.80%
	2–3/week	9	7.60%
	>4/week	3	2.50%
	No Fast Food	66	55.50%
	Missing	1	0.80%
Fresh Vegetable Consumption	No	2	1.70%
	Yes	117	98.30%
Fresh Food Frequency	0 serving/day	1	0.80%
	1–3 servings/day	86	72.30%
	4–5 servings/day	25	21.00%
	At least once/week	1	0.80%
	Missing	6	5.00%
Experience of Stress	No	58	48.70%
	Yes	53	44.50%
	Missing	8	6.70%

## Data Availability

The detailed methods and analyzed data tables are available in Supplementary Material files. The de-identified data from the MMIP cohort used in this study are available on request from Vasantha Padmanabhan.

## Supplementary Materials

The following supporting information can be downloaded at: https://www.mdpi.com/article/10.3390/toxics14050421/s1: Supplementary Methods; Supplementary Tables S1–S12; and Supplementary Figures S1–S3.
