# Supplementary material for: Early Pregnancy Targeted Exposome: Biological Response and Maternal BMI
Source: Toxics. 2026 May 12;14(5):421. doi: 10.3390/toxics14050421 (PMC13211517; doi:10.3390/toxics14050421)
Supplement: Supplementary file 1 [file toxics-14-00421-s001.zip › Supplementary Material Figures S1-S3.pdf]

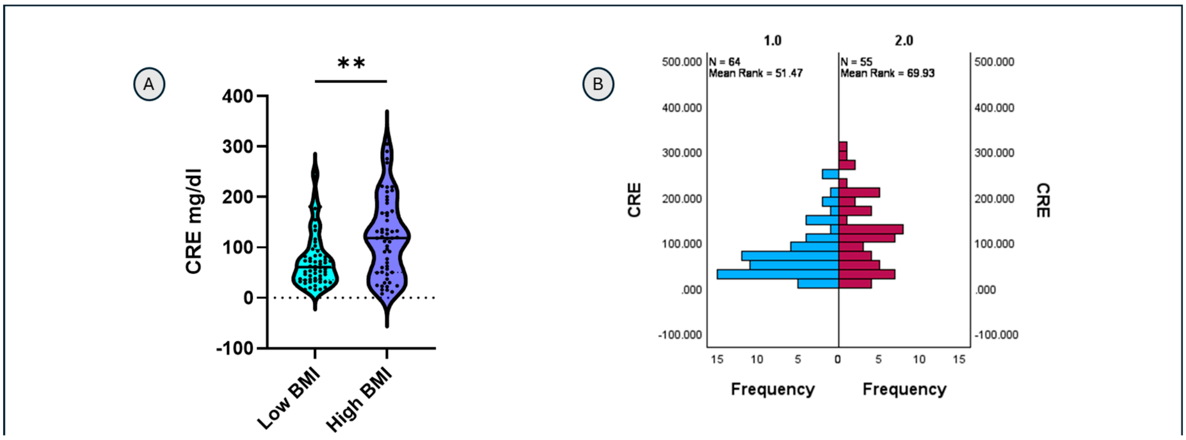

Supplementary Figure S1: Urinary Creatinine levels. Panel A shows urinary creatinine levels mg/dl in low and high BMI groups. Asterisks (**) represent significant based on Mann-Whitney U test (p<0.01). Panel B shows frequency data in low (Blue) and High (Red) BMI groups.

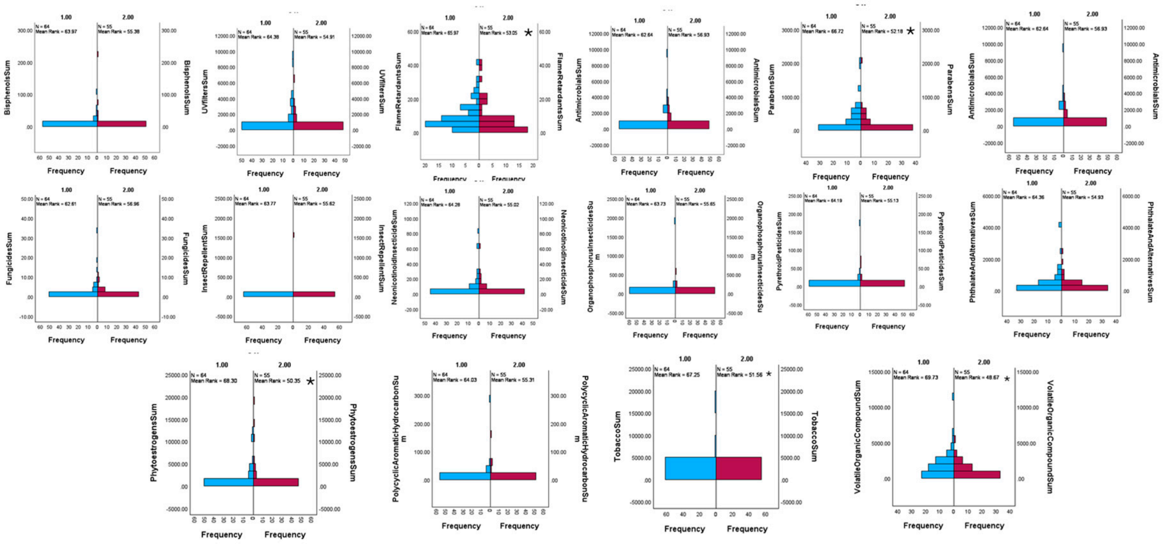

Supplementary Figure S2: Frequency data for Exposure classes before log transformation in low (Blue) and High (Red) BMI group.

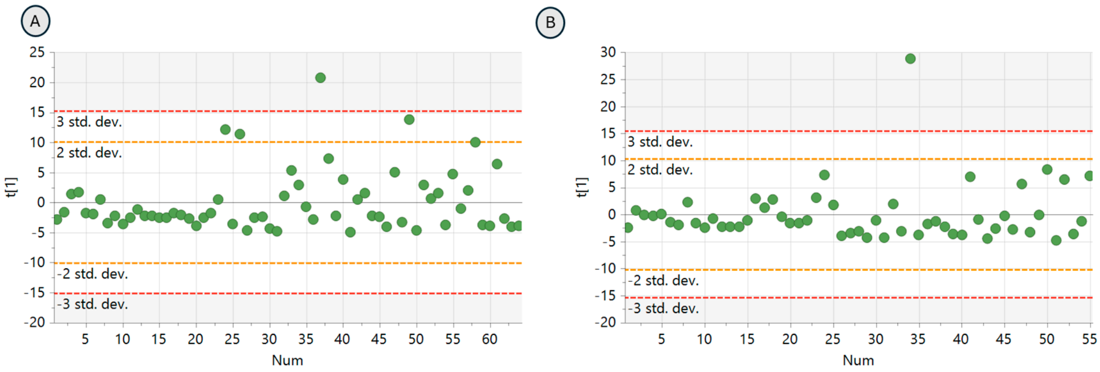

Supplementary Figure S3: Panel A shows outlier in low BMI group and panel B shows outlier in high BMI group.
