# Supplementary material for: Early Pregnancy Targeted Exposome: Biological Response and Maternal BMI
Source: Toxics. 2026 May 12;14(5):421. doi: 10.3390/toxics14050421 (PMC13211517; doi:10.3390/toxics14050421)
Supplement: Supplementary file 1 [file toxics-14-00421-s001.zip › Supplementary Methods.pdf]

# SUPPLEMENTARY METHODS

## Urinary Multi-class LC-MS/MS Assay

### Sample Preparation and Extraction

The investigation utilized an isotope-dilution liquid chromatography-tandem mass spectrometry (LC-MS/MS) technique for quantifying various metabolites and biomarkers, adhering to a previously established protocol (Jagani et al., 2022). This in-depth approach was specifically developed to facilitate the simultaneous analysis of multiple categories of environmental chemicals and their metabolites in a single analytical run.

**Supplementary Table S1** contains a detailed list of all biomarkers of exposure and effect that were part of this study, including their acronyms, CAS numbers, and associated chemical groups.

The sample preparation protocol began with the careful addition of isotopically labeled internal standards to each urine sample, including Carbon-labeled compounds such as $^{13}C_2$, $^{13}C_3$, $^{13}C_4$, $^{13}C_6$, $^{13}C_{12}$, $^{13}C_2{}^{13}C_2$, and various Deuterium-labeled standards ($D_3$, $D_4$, $D_5$, $D_6$, $D_7$, $D_8$, $D_9$, $D_{10}$ and $D_{18}$). This crucial step was essential for ensuring accurate quantification and mitigating any potential matrix effects or losses that might occur during extraction. Utilizing these isotope-labeled internal standards is vital for precise measurement in complex biological matrices like urine, as they behave similarly to native analytes throughout the preparation and analysis processes.

Afterward, the samples underwent an enzymatic deconjugation process using β-glucuronidase and arylsulfatase sourced from *Helix pomatia* (product # 10127060001, Roche Diagnostics via Sigma Aldrich, St. Louis, MO, USA). This enzymatic treatment was key for breaking down glucuronide and sulfate conjugates, releasing parent compounds for further analysis. The deconjugation stage was particularly significant for metabolites that are usually excreted in conjugated forms, such as phenols and phthalate metabolites. The enzyme solution was carefully prepared to maintain optimal activity levels, targeting approximately 100,000 units/mL for β-glucuronidase and 47,500 units/mL for sulfatase. Detailed criteria for enzyme selection and optimization of the hydrolysis process were outlined in the referenced study (Jagani et al., 2022).

Following the enzymatic deconjugation, solid-phase extraction (SPE) was performed using an Oasis HLB hydrophilic-lipophilic balanced reversed-phase 96-well plate, with each well containing 30 mg of sorbent with a particle size of 30 µm (Waters Corporation, Milford, MA). This SPE step aimed to isolate and concentrate the analytes of interest while eliminating potentially interfering substances from the sample matrix. The chosen hydrophilic-lipophilic balanced sorbent was ideal for retaining a broad spectrum of compounds with varying polarities, which is crucial given the diverse range of metabolites and biomarkers investigated in this study. The selection process for the SPE sorbent was thoroughly documented in the referenced methodology (Jagani et al., 2022).

The SPE procedure was meticulously optimized to ensure the efficient extraction of target analytes while minimizing the co-extraction of interfering matrix components. Initially, the SPE plate wells were equilibrated with methanol and conditioned with water to prepare the sorbent for sample loading. The deconjugated urine samples were then introduced onto the SPE plate, allowing the sorbent to retain the analytes while many interfering matrix components were eliminated. Analytes were subsequently eluted from the SPE plate using methanol. The entire SPE process was described comprehensively in the referenced publication (Jagani et al., 2022).

To improve efficiency and reduce the likelihood of manual handling errors, the entire sample preparation process was automated using a liquid handler (epMotion 5075vtc; Eppendorf, Hauppauge, NY, USA). This automation not only enhanced sample throughput but also ensured consistent extraction processes across all samples, thus increasing the overall reliability and reproducibility of the method. The automated system accurately transferred urine samples, added internal standards, and executed all subsequent liquid handling steps with high precision and accuracy.

**Chromatographic Separation**

Chromatographic separation of extracted analytes was an essential element of the analytical method, aimed at resolving intricate mixtures of environmental chemicals and their metabolites. This separation was accomplished with a Sciex Exion UHPLC system (SCIEX, Framingham, MA, USA). To ensure effective separation across the diverse range of compound classes analyzed, a multi-injection strategy was implemented, employing various chromatographic columns and conditions for each injection.

In the first injection, a Hypersil Gold AQ column (3 µm, 4.6 × 150 mm) along with a guard column (3 µm, 4.0 × 10 mm) was used (Thermo Scientific, Waltham, MA, USA). This column was selected for its capability to retain and separate more polar compounds, making it suitable for analyzing pesticide metabolites. The unique stationary phase of the Hypersil Gold AQ column enhanced the retention of polar analytes compared to standard C18 columns.

For the second injection, a Betasil C18 column (5 µm, 2.1 × 100 mm) with a matching guard column (5 µm, 2.1 × 10 mm) was utilized (Thermo Scientific, Waltham, MA, USA). This column was particularly adept at resolving compounds and metabolites related to environmental phenols, polycyclic aromatic hydrocarbons, organophosphate flame retardants, and volatile organic compound metabolites. The C18 stationary phase provided excellent retention and separation for these relatively nonpolar substances.

The third injection employed a Kinetex C8 column (2.6 µm, 150 × 2.1 mm) with a corresponding 2.1 mm guard column (Phenomenex Inc., Torrance, CA, USA). This column offered a different selectivity, optimized for the separation of tobacco smoke metabolites and phthalate metabolites that were not sufficiently resolved by the previous two columns. The C8 stationary phase struck a balance between retaining moderately polar and non-polar compounds.

Each chromatographic method was meticulously optimized regarding mobile phase composition, flow rate, and gradient program to achieve optimal separation for each analyte group. For instance, the method using the Hypersil Gold AQ column featured a mobile phase gradient of 0.1% acetic acid in water (mobile phase A) and a blend of acetonitrile and methanol (1:1) (mobile phase B). The gradient program was designed to begin with a high percentage of the aqueous phase to retain polar compounds while gradually increasing the organic content to elute less polar analytes. The flow rate was set to 0.50 mL/min, providing an optimal balance between chromatographic efficiency and analysis time. The column oven temperature was maintained at 40°C to ensure consistent retention times and peak shapes across all injections. Additionally, to reduce carryover and maintain system cleanliness, the autosampler was programmed to perform extensive rinses between injections using a methanol and water mixture.

**Supplementary Methods Table S2** provides detailed information on the mobile phases, flow rate, and gradient specifications used in the multi-injection technique.

**Mass Spectrometry**

Mass spectrometric detection and quantification of target analytes were conducted using a Sciex 6500+ triple quadrupole mass spectrometer with an electrospray ionization (ESI) source (SCIEX, Framingham, MA, USA). This instrument was chosen for its exceptional sensitivity, broad dynamic range, and capability for rapid polarity switching, which was crucial for the multi-class analysis undertaken.

The mass spectrometer operated in multiple reaction monitoring (MRM) mode, which offered enhanced selectivity and sensitivity for targeted analyses. During MRM mode, specific transitions from precursor ions to product ions were tracked for each analyte, utilizing the most intense transition for quantification and a secondary transition for confirmation. This methodology not only improved specificity but also increased the signal-to-noise ratio, resulting in lower detection and quantification limits.

The ESI source functioned in both positive and negative ionization modes, either in succession or simultaneously, depending on the analytes measured in each injection. This adaptability in ionization polarity facilitated optimal detection of a diverse array of compounds with varying chemical characteristics. Ion source parameters were meticulously fine-tuned to maximize sensitivity across all analyte categories. The curtain gas flow, which blocks neutrals from entering the mass analyzer, was maintained at 30 psi to promote effective desolvation. The nebulizer gas (ion source gas 1) and heater gas (ion source gas 2) were set to 50 psi and 55 psi, respectively, to enhance ionization efficiency. Ion source voltage was adjusted to 4500 V for positive ESI mode and -4500 V for negative ESI mode. The source temperature was kept at 400°C to ensure complete desolvation of the LC eluent.

For each analyte, specific parameters such as declustering potential, entrance potential, collision energy, and collision cell exit potential were individually optimized. This optimization process

involved directly infusing each compound into the mass spectrometer and systematically adjusting these parameters to achieve the highest signal intensity for both quantifier and qualifier ion transitions.

Optimized instrumentation parameters for each native and labeled analyte, including SPE extraction and LC injection numbers, LC column specifications, compound retention time (RT), and mass spectrometry parameters such as MRM transition, declustering potential (DP), entrance potential (EP), collision energy (CE), and cell exit potential (CXP), can be found in **Supplementary Methods Table S3**.

**Method Validation**

A thorough validation of the method was conducted to confirm the analytical technique's reliability and robustness. This validation involved determining the limits of detection (LOD) and quantification (LOQ), assessing linearity across a broad concentration spectrum, evaluating accuracy and precision, and examining possible matrix effects.

The LOD and LOQ were established using a statistical method based on the analysis of low-level spiked samples. Specifically, matrix blanks of synthetic urine were spiked with native standards at a concentration of 1.0 ng/mL and analyzed in replicate (n=10). The LOD was determined as three times the standard deviation of these replicate measurements, while the LOQ was defined as ten times the standard deviation. This methodology offered a realistic evaluation of the method's performance within the matrix, considering both instrumental noise and potential interferences from the matrix. The limits of detection for the analytes studied can be found in **Supplementary Methods Table S4.**

Linearity was assessed by analyzing calibration standards prepared in matrix-matched samples across a concentration range of 0.1 to 100 ng/mL. Calibration curves were created by plotting the ratio of the analyte's peak area to that of the corresponding isotope-labeled internal standard against the spiked concentration. A weighting factor of 1/x was utilized in the regression to address heteroscedasticity within the extensive concentration range. Acceptable linearity was defined as a coefficient of determination ($r^2$) exceeding 0.99 for each analyte.

To evaluate the accuracy and precision of the method, quality control (QC) samples were prepared at various concentration levels throughout the calibration range. These QC samples included procedural blanks, matrix blanks, and matrix-matched samples spiked at low (unspiked QC urine pool), medium (QC urine pool spiked with 1 ng/mL of native analyte standards mixture), and high (QC urine pool spiked with 10 ng/mL of native analyte standards mixture) concentrations. The QC samples were analyzed alongside each batch of unknown samples to monitor the method's performance and ensure the validity of the analytical results.

**Supplementary Methods Table S5** presents the characterized analyte concentrations in QC urine pools. It highlights the results from triplicate analyses of spiked samples conducted at two concentration levels (1 and 10 ng/mL) over five distinct analytical batches.

**Quality Assurance and External Validation**

To maintain consistent method performance and ensure comparability with other laboratories, the analytical team engaged in proficiency testing programs run by established external quality assessment organizations. These included the German External Quality Assessment Scheme (G-EQUAS), which focuses on analyses of biological materials (http://www.g-equas.de/), and the Organic Substances in Urine Quality Assessment Scheme (OSEQAS), managed by the Centre de Toxicologie du Quebec (CTQ) (https://www.inspq.qc.ca/en/ctq/eqas/oqesas/description). Successful involvement in these initiatives confirmed the accuracy and dependability of the developed method for quantifying environmental chemical biomarkers in urine.

The Mount Sinai laboratory is affiliated with the NIEHS's Children's Health Exposure Analysis Resource (CHEAR) consortium, which emphasizes exposure analysis through both conventional biomonitoring techniques and untargeted analysis of the exposome. This consortium has evolved into the Human Health Exposure Analysis Resource (HHEAR) (https://hhearprogram.org/). The Mount Sinai laboratory adheres to the QA/QC and method validation protocols established by the CHEAR and HHEAR programs as detailed in Kannan et al. (2021).

**Supplementary Methods Text References:**

K. Kannan, A. Stathis, M.J. Mazzella, S.S. Andra, D.B. Barr, S.S. Hecht, L.S. Merrill, A.L. Galusha, P.J. Parsons, Quality assurance and harmonization for targeted biomonitoring measurements of environmental organic chemicals across the Children's Health Exposure Analysis Resource laboratory network, Int J Hyg Environ Health 234 (2021) 113741. https://doi.org/10.1016/j.ijheh.2021.113741.

R. Jagani, D. Pulivarthi, D. Patel, R.J. Wright, R.O. Wright, M. Arora, M.S. Wolff, S.S. Andra, Validated single urinary assay designed for exposomic multi-class biomarkers of common environmental exposures, Anal Bioanal Chem 414(19) (2022) 5943-5966. https://doi.org/10.1007/s00216-022-04159-4.

**Supplementary Methods Table S2**. Liquid chromatography parameters utilized in the multi-class urine assay conducted in this study.

| | **Injection 1, Extract 1** | | | **Injection 2, Extract 1** | | | **Injection 3, Extract 2** | | |
|---|---|---|---|---|---|---|---|---|---|
| LC column | Hypersil Gold AQ | | | Betasil C18 | | | Kinetex C8 | | |
| Mobile phase A | 0.1% acetic acid in water-optima LCMS grade. | | | Water-optima LCMS grade. | | | 0.1% formic acid in water-optima LCMS grade. | | |
| Mobile phase B | Acetonitrile: methanol (1:1)-optima LCMS grade. | | | Acetonitrile-optima LCMS grade. | | | 0.1% formic acid in acetonitrile-optima LCMS grade. | | |
| Gradient program | Time (min) | Flow rate (mL/min) | %B | Time (min) | Flow rate (mL/min) | %B | Time (min) | Flow rate (mL/min) | %B |
| | 0.00 | 0.50 | 5 | 0.00 | 0.20 | 1 | 0.00 | 0.15 | 5 |
| | 1.00 | 0.50 | 5 | 2.00 | 0.20 | 1 | 2.00 | 0.15 | 5 |
| | 6.00 | 0.50 | 50 | 3.00 | 0.20 | 5 | 11.00 | 0.30 | 50 |
| | 8.00 | 0.50 | 50 | 4.00 | 0.50 | 5 | 11.50 | 0.30 | 95 |
| | 11.00 | 0.50 | 95 | 5.00 | 0.50 | 50 | 13.50 | 0.30 | 95 |
| | 14.00 | 0.50 | 95 | 7.00 | 0.50 | 50 | 14.00 | 0.15 | 5 |
| | 15.00 | 0.50 | 5 | 9.00 | 0.50 | 99 | 18.00 | 0.15 | 5 |
| | 18.00 | 0.50 | 5 | 10.50 | 0.50 | 99 | | | |
| | | | | 11.00 | 0.20 | 99 | | | |
| | | | | 12.00 | 0.20 | 1 | | | |
| | | | | 18.00 | 0.20 | 1 | | | |

**Supplementary Methods Table S3**. Summary of native analytes and their corresponding labeled internal standards employed in this study, including details of SPE extraction, LC injection number, LC column specifications, compound retention times (RT), and mass spectrometry parameters such as MRM transition, declustering potential (DP), entrance potential (EP), collision energy (CE), and cell exit potential (CXP).

| Analyte # | Analyte Code | Extract # | Injection # | LC column | RT (min) | MS ionization mode | Precursor ion (m/z) | Quantifier product ion (m/z) | Dwell time (ms) | DP (V) | EP (V) | CE (eV) | CXP (V) |
|---|---|---|---|---|---|---|---|---|---|---|---|---|---|
| **Organophosphate Flame Retardant Metabolites** | | | | | | | | | | | | | |
| 1 | BCETP | 1 | 2 | Betasil | 5.48 | ESI Neg | 221.0 | 35.0 | 20 | -30 | -10 | -32 | -10 |
| | BCETP-$d_8$ | 1 | 2 | Betasil | 5.48 | ESI Neg | 229.0 | 35.0 | 20 | -30 | -10 | -37 | -10 |
| 2 | BDCPP | 1 | 2 | Betasil | 6.05 | ESI Neg | 319.0 | 35.0 | 20 | -25 | -10 | -40 | -10 |
| | BDCPP-$d_{10}$ | 1 | 2 | Betasil | 6.05 | ESI Neg | 329.0 | 35.0 | 20 | -30 | -10 | -45 | -10 |
| 3 | DBUP | 1 | 2 | Betasil | 5.84 | ESI Neg | 208.9 | 78.7 | 20 | -50 | -10 | -20 | -21 |
| | DBUP-$d_{18}$ | 1 | 2 | Betasil | 5.84 | ESI Neg | 227.0 | 78.8 | 20 | -50 | -10 | -50 | -10 |
| 4 | DPHP | 1 | 2 | Betasil | 5.90 | ESI Neg | 249.0 | 93.0 | 20 | -50 | -10 | -33 | -15 |
| | DPHP-$d_{10}$ | 1 | 2 | Betasil | 5.90 | ESI Neg | 259.0 | 98.0 | 20 | -50 | -10 | -40 | -15 |
| **Oxidative Stress Biomarkers** | | | | | | | | | | | | | |
| 5 | F2A8IP | 1 | 3 | Kinetex | 10.92 | ESI Neg | 353.0 | 193.1 | 10 | -175 | -10 | -36 | -9 |
| | F2A8IP -$d_4$ | 1 | 3 | Kinetex | 10.92 | ESI Neg | 357.2 | 313.2 | 10 | -55 | -10 | -28 | -13 |
| 6 | HNEMA | 1 | 3 | Kinetex | 9.84 | ESI Neg | 318.0 | 161.9 | 10 | -40 | -10 | -16 | -19 |
| | HNEMA-$d_3$ | 1 | 3 | Kinetex | 9.84 | ESI Neg | 321.0 | 165.0 | 10 | -40 | -10 | -16 | -17 |
| **Personal Care and Consumer Product Chemicals and Metabolites** | | | | | | | | | | | | | |
| 7 | BP1 | 1 | 2 | Betasil | 6.83 | ESI Neg | 213.0 | 91.0 | 20 | -60 | -10 | -38 | -11 |
| | BP1-$d_5$ | 1 | 2 | Betasil | 6.83 | ESI Neg | 218.0 | 91.0 | 20 | -60 | -10 | -38 | -11 |
| 8 | BP3 | 1 | 2 | Betasil | 8.64 | ESI Neg | 227.0 | 211.0 | 10 | -60 | -10 | -38 | -11 |
| | BP3-$^{13}C_6$ | 1 | 2 | Betasil | 8.64 | ESI Neg | 232.9 | 216.9 | 10 | -60 | -10 | -32 | -25 |
| 9 | BPA | 1 | 2 | Betasil | 6.46 | ESI Neg | 227.1 | 133.0 | 20 | -30 | -10 | -30 | -16 |
| | BPA-$^{13}C_{12}$ | 1 | 2 | Betasil | 6.46 | ESI Neg | 239.0 | 224.0 | 20 | -25 | -15 | -25 | -16 |
| 10 | BPS | 1 | 2 | Betasil | 5.79 | ESI Neg | 249.1 | 108.0 | 20 | -80 | -10 | -35 | -21 |
| | BPS-$^{13}C_{12}$ | 1 | 2 | Betasil | 5.79 | ESI Neg | 261.0 | 114.0 | 20 | -40 | -10 | -30 | -11 |
| 11 | BPZ | 1 | 2 | Betasil | 7.67 | ESI Neg | 267.0 | 173.0 | 10 | -30 | -10 | -35 | -21 |
| | BPZ-$d_6$ | 1 | 2 | Betasil | 7.67 | ESI Neg | 273.0 | 179.0 | 10 | -30 | -10 | -35 | -21 |
| 12 | BUPB | 1 | 2 | Betasil | 7.26 | ESI Neg | 193.0 | 92.0 | 10 | -90 | -10 | -30 | -11 |
| | BUPB-$^{13}C_6$ | 1 | 2 | Betasil | 7.26 | ESI Neg | 199.0 | 98.0 | 10 | -40 | -10 | -30 | -11 |
| 13 | ETPB | 1 | 2 | Betasil | 6.16 | ESI Neg | 165.0 | 92.0 | 20 | -60 | -10 | -28 | -11 |
| | ETPB-$^{13}C_6$ | 1 | 2 | Betasil | 6.16 | ESI Neg | 171.0 | 98.0 | 20 | -40 | -10 | -30 | -11 |
| 14 | MEPB | 1 | 2 | Betasil | 5.90 | ESI Neg | 151.0 | 92.0 | 20 | -60 | -10 | -30 | -11 |
| | MEPB-$^{13}C_6$ | 1 | 2 | Betasil | 5.90 | ESI Neg | 157.0 | 98.0 | 20 | -60 | -10 | -30 | -11 |
| 15 | PRPB | 1 | 2 | Betasil | 6.58 | ESI Neg | 179.0 | 92.0 | 20 | -50 | -10 | -28 | -11 |
| | PRPB-$^{13}C_6$ | 1 | 2 | Betasil | 6.58 | ESI Neg | 185.0 | 98.0 | 20 | -40 | -10 | -30 | -11 |
| 16 | TCC | 1 | 2 | Betasil | 9.00 | ESI Neg | 312.8 | 160.0 | 10 | -60 | -10 | -18 | -13 |
| | TCC-$^{13}C_6$ | 1 | 2 | Betasil | 9.00 | ESI Neg | 318.8 | 159.9 | 10 | -75 | -10 | -18 | -11 |
| 17 | TCS | 1 | 2 | Betasil | 9.00 | ESI Neg | 287.0 | 35.0 | 10 | -15 | -10 | -35 | -16 |
| | TCS-$^{13}C_{12}$ | 1 | 2 | Betasil | 9.00 | ESI Neg | 299.0 | 35.0 | 10 | -60 | -10 | -35 | -3 |
| **Pesticides and Metabolites** | | | | | | | | | | | | | |
| 18 | ACE | 1 | 1 | Hypersil | 10.29 | ESI Pos | 222.8 | 126.0 | 10 | 30 | 10 | 23 | 10 |
| | ACE-$d_3$ | 1 | 1 | Hypersil | 10.29 | ESI Pos | 226.0 | 126.0 | 10 | 30 | 10 | 32 | 7 |

| Analyte # | Analyte Code | Extract # | Injection # | LC column | RT (min) | MS ionization mode | Precursor ion (m/z) | Quantifier product ion (m/z) | Dwell time (ms) | DP (V) | EP (V) | CE (eV) | CXP (V) |
|---|---|---|---|---|---|---|---|---|---|---|---|---|---|
| 19 | CDCCA | 1 | 1 | Hypersil | 14.28 | ESI Neg | 207.0 | 35.0 | 10 | -35 | -10 | -28 | -5 |
| | CDCCA -$^{13}C_2$ | 1 | 1 | Hypersil | 14.28 | ESI Neg | 210.0 | 35.0 | 10 | -70 | -10 | -32 | -5 |
| 20 | CINA6 | 1 | 1 | Hypersil | 9.28 | ESI Neg | 155.9 | 111.9 | 10 | -35 | -10 | -14 | -13 |
| | CINA6-$^{13}C_6$ | 1 | 1 | Hypersil | 9.28 | ESI Neg | 161.9 | 116.9 | 10 | -5 | -10 | -16 | -11 |
| 21 | CLOT | 1 | 1 | Hypersil | 9.76 | ESI Pos | 250.0 | 169.1 | 10 | 66 | 10 | 19 | 10 |
| | CLOT-$d_3$ | 1 | 1 | Hypersil | 9.76 | ESI Pos | 252.9 | 172.1 | 10 | 66 | 10 | 19 | 10 |
| 22 | D24 | 1 | 1 | Hypersil | 13.00 | ESI Neg | 218.8 | 160.8 | 10 | -45 | -10 | -18 | -27 |
| | D24-$^{13}C_6$ | 1 | 1 | Hypersil | 13.00 | ESI Neg | 224.8 | 166.9 | 10 | -70 | -10 | -20 | -15 |
| 23 | DCBA | 1 | 1 | Hypersil | 10.69 | ESI Pos | 222.0 | 149.0 | 10 | 30 | 10 | 23 | 4 |
| | DCBA-$d_{10}$ | 1 | 1 | Hypersil | 10.60 | ESI Pos | 232.0 | 149.0 | 10 | 30 | 10 | 25 | 4 |
| 24 | DEDP | 1 | 1 | Hypersil | 4.56 | ESI Neg | 185.0 | 111.0 | 10 | -70 | -10 | -20 | -21 |
| | DEDP-$d_{10}$ | 1 | 1 | Hypersil | 4.56 | ESI Neg | 195.0 | 111.0 | 10 | -70 | -10 | -20 | -10 |
| 25 | DEP | 1 | 1 | Hypersil | 4.56 | ESI Neg | 153.0 | 125.0 | 10 | -70 | -10 | -20 | -21 |
| | DEP-$d_{10}$ | 1 | 1 | Hypersil | 4.56 | ESI Neg | 163.0 | 79.0 | 10 | -70 | -10 | -30 | -10 |
| 26 | DETP | 1 | 1 | Hypersil | 4.56 | ESI Neg | 169.0 | 141.0 | 10 | -70 | -10 | -20 | -21 |
| | DETP-$d_{10}$ | 1 | 1 | Hypersil | 4.56 | ESI Neg | 179.0 | 95.0 | 10 | -70 | -10 | -20 | -10 |
| 27 | DMDP | 1 | 1 | Hypersil | 4.10 | ESI Neg | 157.0 | 142.0 | 10 | -70 | -10 | -20 | -21 |
| | DMDP-$d_6$ | 1 | 1 | Hypersil | 4.10 | ESI Neg | 163.0 | 145.0 | 10 | -70 | -10 | -30 | -10 |
| 28 | DMP | 1 | 1 | Hypersil | 3.40 | ESI Neg | 125.0 | 110.0 | 10 | -70 | -10 | -20 | -21 |
| | DMP-$d_6$ | 1 | 1 | Hypersil | 3.40 | ESI Neg | 131.0 | 79.0 | 10 | -70 | -10 | -30 | -10 |
| 29 | DMTP | 1 | 1 | Hypersil | 3.70 | ESI Neg | 141.0 | 126.0 | 10 | -70 | -10 | -20 | -21 |
| | DMTP-$d_6$ | 1 | 1 | Hypersil | 3.70 | ESI Neg | 147.0 | 97.0 | 10 | -70 | -10 | -30 | -10 |
| 30 | ECBA | 1 | 1 | Hypersil | 9.22 | ESI Pos | 194.1 | 149.0 | 10 | 51 | 10 | 25 | 16 |
| | ECBA-$d_5$ | 1 | 1 | Hypersil | 9.25 | ESI Pos | 199.1 | 149.9 | 10 | 51 | 10 | 25 | 10 |
| 31 | IMI | 1 | 1 | Hypersil | 9.87 | ESI Pos | 256.0 | 209.1 | 10 | 41 | 10 | 23 | 12 |
| | IMI-$d_4$ | 1 | 1 | Hypersil | 9.87 | ESI Pos | 261.0 | 214.2 | 10 | 16 | 10 | 25 | 10 |
| 32 | IMPY | 1 | 1 | Hypersil | 8.73 | ESI Pos | 153.1 | 84.0 | 10 | 30 | 10 | 23 | 10 |
| | IMPY-$^{13}C_4$ | 1 | 1 | Hypersil | 8.73 | ESI Pos | 157.1 | 88.0 | 10 | 56 | 10 | 25 | 10 |
| 33 | MDA | 1 | 1 | Hypersil | 9.79 | ESI Neg | 273.0 | 140.9 | 10 | -50 | -10 | -14 | -15 |
| | MDA-$d_6$ | 1 | 1 | Hypersil | 9.79 | ESI Neg | 276.8 | 140.9 | 10 | -50 | -10 | -14 | -15 |
| 34 | NDMA | 1 | 1 | Hypersil | 9.91 | ESI Neg | 206.9 | 41.0 | 10 | -60 | -10 | -44 | -19 |
| | NDMA-$^{13}C_3$ | 1 | 1 | Hypersil | 9.91 | ESI Neg | 209.9 | 41.0 | 10 | -75 | -10 | -48 | -19 |
| 35 | NDMT | 1 | 1 | Hypersil | 10.13 | ESI Pos | 278.0 | 132.0 | 10 | 50 | 10 | 25 | 10 |
| | DCBA-$d_{10}$ | 1 | 1 | Hypersil | 10.60 | ESI Pos | 232.0 | 149.0 | 10 | 30 | 10 | 25 | 4 |
| 36 | NIT | 1 | 1 | Hypersil | 9.91 | ESI Pos | 271.1 | 126.1 | 10 | 10 | 10 | 40 | 10 |
| | NIT-$d_3$ | 1 | 1 | Hypersil | 9.91 | ESI Pos | 274.1 | 228.0 | 10 | 11 | 10 | 17 | 12 |
| 37 | OFIMI | 1 | 1 | Hypersil | 9.11 | ESI Pos | 254.1 | 206.0 | 10 | 41 | 10 | 19 | 12 |
| | ECBA-$d_5$ | 1 | 1 | Hypersil | 9.25 | ESI Pos | 199.1 | 149.0 | 10 | 51 | 10 | 25 | 10 |
| 38 | OHIMI | 1 | 1 | Hypersil | 9.28 | ESI Pos | 272.0 | 225.0 | 10 | 30 | 10 | 25 | 10 |
| | THX-$d_3$ | 1 | 1 | Hypersil | 9.14 | ESI Pos | 294.9 | 214.1 | 10 | 41 | 10 | 19 | 12 |
| 39 | OHTBZ | 1 | 1 | Hypersil | 11.43 | ESI Pos | 218.0 | 191.2 | 10 | 141 | 10 | 37 | 24 |
| | THI-$d_4$ | 1 | 1 | Hypersil | 10.94 | ESI Pos | 257.1 | 126.0 | 10 | 66 | 10 | 29 | 14 |
| 40 | PBA | 1 | 1 | Hypersil | 14.10 | ESI Neg | 213.0 | 93.0 | 10 | -60 | -10 | -38 | -8 |
| | PBA-$^{13}C_6$ | 1 | 1 | Hypersil | 14.10 | ESI Neg | 219.0 | 99.0 | 10 | -60 | -10 | -38 | -8 |
| 41 | PCP | 1 | 1 | Hypersil | 15.55 | ESI Neg | 265.0 | 35.0 | 10 | -60 | -10 | -38 | -16 |

| Analyte # | Analyte Code | Extract # | Injection # | LC column | RT (min) | MS ionization mode | Precursor ion (m/z) | Quantifier product ion (m/z) | Dwell time (ms) | DP (V) | EP (V) | CE (eV) | CXP (V) |
|---|---|---|---|---|---|---|---|---|---|---|---|---|---|
| | PCP-$^{13}C_6$ | 1 | 1 | Hypersil | 15.55 | ESI Neg | 270.7 | 34.9 | 10 | -100 | -10 | -56 | -17 |
| 42 | PNP | 1 | 1 | Hypersil | 10.91 | ESI Neg | 138.0 | 108.0 | 10 | -50 | -10 | -38 | -16 |
| | PNP-$^{13}C_6$ | 1 | 1 | Hypersil | 10.91 | ESI Neg | 144.0 | 114.0 | 10 | -50 | -10 | -38 | -16 |
| 43 | TCP | 1 | 1 | Hypersil | 13.82 | ESI Neg | 195.8 | 35.0 | 10 | -5 | -10 | -42 | -5 |
| | TCP-$^{13}C_3$ | 1 | 1 | Hypersil | 13.82 | ESI Neg | 198.8 | 35.0 | 10 | -45 | -10 | -42 | -5 |
| 44 | TDCCA | 1 | 1 | Hypersil | 14.07 | ESI Neg | 209.0 | 35.0 | 10 | -60 | -10 | -40 | -5 |
| | TDCCA-$^{13}C_2$ | 1 | 1 | Hypersil | 14.07 | ESI Neg | 210.0 | 35.0 | 10 | -30 | -10 | -38 | -1 |
| 45 | THPI | 1 | 1 | Hypersil | 8.89 | ESI Neg | 150.0 | 96.0 | 10 | -50 | -10 | -25 | -15 |
| | CINA6-$^{13}C_6$ | 1 | 1 | Hypersil | 9.28 | ESI Neg | 161.9 | 116.9 | 10 | -5 | -10 | -16 | -11 |
| **Phthalate and Phthalate Alternative Metabolites** | | | | | | | | | | | | | |
| 46 | MBZP | 1 | 3 | Kinetex | 12.07 | ESI Neg | 255.2 | 76.9 | 10 | -50 | -10 | -33 | -37 |
| | MBZP-$^{13}C_2{}^{13}C_2$ | 1 | 3 | Kinetex | 12.07 | ESI Neg | 259.1 | 185.1 | 10 | -50 | -10 | -15 | -15 |
| 47 | MCINP | 1 | 3 | Kinetex | 12.53 | ESI Neg | 335.1 | 187.1 | 10 | -50 | -10 | -12 | -15 |
| | MCINP-$^{13}C_2{}^{13}C_2$ | 1 | 3 | Kinetex | 12.53 | ESI Neg | 339.1 | 187.1 | 10 | -50 | -10 | -12 | -15 |
| 48 | MCIOP | 1 | 3 | Kinetex | 11.87 | ESI Neg | 321.1 | 173.1 | 10 | -50 | -10 | -12 | -15 |
| | MCIOP-$^{13}C_2{}^{13}C_2$ | 1 | 3 | Kinetex | 11..87 | ESI Neg | 325.1 | 173.1 | 10 | -50 | -10 | -12 | -15 |
| 49 | MCOCH | 1 | 3 | Kinetex | 12.44 | ESI Neg | 327.1 | 173.0 | 10 | -50 | -10 | -24 | -9 |
| | MCOCH -$^{13}C_4$ | 1 | 3 | Kinetex | 12.44 | ESI Neg | 331.0 | 173.0 | 10 | -50 | -10 | -12 | -15 |
| 50 | MCPP | 1 | 3 | Kinetex | 9.10 | ESI Neg | 251.1 | 120.9 | 10 | -50 | -10 | -26 | -13 |
| | MCPP-$^{13}C_2{}^{13}C_2$ | 1 | 3 | Kinetex | 9.10 | ESI Neg | 255.1 | 103.1 | 10 | -50 | -10 | -8 | -15 |
| 51 | MECPP | 1 | 3 | Kinetex | 11.35 | ESI Neg | 307.1 | 159.1 | 10 | -50 | -10 | -15 | -15 |
| | MECPP-$^{13}C_4$ | 1 | 3 | Kinetex | 11.35 | ESI Neg | 311.1 | 159.1 | 10 | -50 | -10 | -15 | -15 |
| 52 | MECPTP | 1 | 3 | Kinetex | 11.80 | ESI Neg | 307.4 | 121.1 | 10 | -50 | -10 | -18 | -15 |
| | MECPTP-$d_4$ | 1 | 3 | Kinetex | 11.80 | ESI Neg | 311.4 | 168.9 | 10 | -50 | -10 | -18 | -15 |
| 53 | MEHHP | 1 | 3 | Kinetex | 11.59 | ESI Neg | 293.1 | 121.1 | 10 | -50 | -10 | -18 | -15 |
| | MEHHP-$^{13}C_4$ | 1 | 3 | Kinetex | 11.59 | ESI Neg | 297.1 | 145.1 | 10 | -50 | -10 | -18 | -15 |
| 54 | MEHHTP | 1 | 3 | Kinetex | 11.91 | ESI Neg | 293.1 | 120.9 | 10 | -50 | -10 | -24 | -12 |
| | MEHHTP-$d_4$ | 1 | 3 | Kinetex | 11.91 | ESI Neg | 297.1 | 125.1 | 10 | -50 | -10 | -18 | -15 |
| 55 | MEHP | 1 | 3 | Kinetex | 13.06 | ESI Neg | 277.2 | 134.0 | 10 | -50 | -10 | -21 | -7 |
| | MEHP-$^{13}C_2{}^{13}C_2$ | 1 | 3 | Kinetex | 13.06 | ESI Neg | 281.1 | 137.1 | 10 | -50 | -10 | -13 | -15 |
| 56 | MEHTP | 1 | 3 | Kinetex | 13.20 | ESI Neg | 277.1 | 233.1 | 10 | -50 | -10 | -13 | -15 |
| | MEHTP-$^{13}C_6$ | 1 | 3 | Kinetex | 13.20 | ESI Neg | 283.1 | 239.1 | 10 | -50 | -10 | -13 | -15 |
| 57 | MEOHP | 1 | 3 | Kinetex | 11.78 | ESI Neg | 291.1 | 121.1 | 10 | -50 | -10 | -18 | -15 |
| | MEOHP-$^{13}C_4$ | 1 | 3 | Kinetex | 11.78 | ESI Neg | 295.1 | 124.1 | 10 | -50 | -10 | -18 | -15 |
| 58 | MEOHTP | 1 | 3 | Kinetex | 12.27 | ESI Neg | 291.1 | 121.1 | 10 | -50 | -10 | -18 | -15 |
| | MEHHTP-$d_4$ | 1 | 3 | Kinetex | 11.91 | ESI Neg | 297.1 | 125.1 | 10 | -50 | -10 | -18 | -15 |
| 59 | MEP | 1 | 3 | Kinetex | 9.93 | ESI Neg | 193.0 | 77.0 | 10 | -50 | -10 | -19 | -13 |
| | MEP-$^{13}C_2{}^{13}C_2$ | 1 | 3 | Kinetex | 9.93 | ESI Neg | 197.1 | 79.1 | 10 | -50 | -10 | -15 | -15 |
| 60 | MHNCH | 1 | 3 | Kinetex | 12.60 | ESI Neg | 313.1 | 153.1 | 10 | -50 | -10 | -12 | -15 |
| | MHNCH -$^{13}C_4$ | 1 | 3 | Kinetex | 12.60 | ESI Neg | 317.1 | 157.0 | 10 | -50 | -10 | -12 | -15 |
| 61 | MIBP | 1 | 3 | Kinetex | 11.85 | ESI Neg | 221.1 | 76.9 | 10 | -50 | -10 | -23 | -8 |
| | MIBP-$^{13}C_2{}^{13}C_2$ | 1 | 3 | Kinetex | 11.85 | ESI Neg | 225.1 | 79.1 | 10 | -50 | -10 | -18 | -15 |
| 62 | MMP | 1 | 3 | Kinetex | 9.06 | ESI Neg | 179.0 | 77.0 | 10 | -50 | -10 | -26 | -9 |
| | MMP-$^{13}C_2{}^{13}C_2$ | 1 | 3 | Kinetex | 9.06 | ESI Neg | 183.1 | 79.1 | 10 | -50 | -10 | -20 | -15 |
| 63 | MNBP | 1 | 3 | Kinetex | 11.85 | ESI Neg | 221.1 | 76.9 | 10 | -50 | -10 | -25 | -10 |

| Analyte # | Analyte Code | Extract # | Injection # | LC column | RT (min) | MS ionization mode | Precursor ion (m/z) | Quantifier product ion (m/z) | Dwell time (ms) | DP (V) | EP (V) | CE (eV) | CXP (V) |
|---|---|---|---|---|---|---|---|---|---|---|---|---|---|
| | MNBP-$^{13}C_2{}^{13}C_2$ | 1 | 3 | Kinetex | 11.85 | ESI Neg | 225.1 | 79.1 | 10 | -50 | -10 | -18 | -15 |
| 64 | MONCH | 1 | 3 | Kinetex | 12.70 | ESI Neg | 311.1 | 153.0 | 10 | -50 | -10 | -25 | -17 |
| | MONCH-$^{13}C_4$ | 1 | 3 | Kinetex | 12.70 | ESI Neg | 315.0 | 157.0 | 10 | -50 | -10 | -12 | -15 |
| 65 | MONP | 1 | 3 | Kinetex | 12.27 | ESI Neg | 305.0 | 121.0 | 10 | -50 | -10 | -24 | -7 |
| | MONP-$^{13}C_2{}^{13}C_2$ | 1 | 3 | Kinetex | 12.27 | ESI Neg | 309.0 | 124.0 | 10 | -50 | -10 | -24 | -8 |
| 66 | MPCHP | 1 | 3 | Kinetex | 12.52 | ESI Neg | 335.1 | 187.0 | 10 | -50 | -10 | -12 | -15 |
| | MPCHP-$^{13}C_2{}^{13}C_2$ | 1 | 3 | Kinetex | 12.52 | ESI Neg | 339.1 | 187.1 | 10 | -50 | -10 | -12 | -15 |
| 67 | MPHHP | 1 | 3 | Kinetex | 12.69 | ESI Neg | 321.0 | 121.0 | 10 | -50 | -10 | -25 | -6 |
| | MPHHP-$^{13}C_2{}^{13}C_2$ | 1 | 3 | Kinetex | 12.69 | ESI Neg | 325.0 | 124.0 | 10 | -50 | -10 | -26 | -7 |
| 68 | MPOHP | 1 | 3 | Kinetex | 12.73 | ESI Neg | 319.0 | 121.0 | 10 | -50 | -10 | -28 | -6 |
| | MPOHP-$^{13}C_2{}^{13}C_2$ | 1 | 3 | Kinetex | 12.73 | ESI Neg | 323.0 | 124.0 | 10 | -50 | -10 | -24 | -7 |
| **Phytoestrogens and Metabolites** | | | | | | | | | | | | | |
| 69 | DAZ | 1 | 3 | Kinetex | 9.92 | ESI Neg | 252.9 | 132.0 | 10 | -180 | -10 | -56 | -13 |
| | DAZ-$d_4$ | 1 | 3 | Kinetex | 9.91 | ESI Neg | 256.9 | 135.9 | 10 | -185 | -10 | -54 | -13 |
| 70 | EQU | 1 | 3 | Kinetex | 10.92 | ESI Neg | 240.9 | 121.0 | 10 | -160 | -10 | -22 | -11 |
| | F2A8IP-$d_4$ | 1 | 3 | Kinetex | 10.92 | ESI Neg | 357.2 | 313.2 | 10 | -55 | -10 | -28 | -13 |
| 71 | ETL | 1 | 3 | Kinetex | 11.09 | ESI Neg | 297.0 | 253.1 | 10 | -160 | -10 | -30 | -11 |
| | F2A8IP-$d_4$ | 1 | 3 | Kinetex | 10.92 | ESI Neg | 357.2 | 313.2 | 10 | -55 | -10 | -28 | -13 |
| 72 | GNS | 1 | 3 | Kinetex | 10.90 | ESI Neg | 268.9 | 132.9 | 10 | -170 | -10 | -42 | -13 |
| | GNS-$d_4$ | 1 | 3 | Kinetex | 10.90 | ESI Neg | 272.9 | 137.0 | 10 | -185 | -10 | -42 | -13 |
| **Polycyclic Aromatic Hydrocarbon (PAH) Metabolites** | | | | | | | | | | | | | |
| 73 | FLUO2 | 1 | 2 | Betasil | 7.28 | ESI Neg | 181.0 | 180.0 | 10 | -15 | -10 | -30 | -16 |
| | FLUO2-$d_9$ | 1 | 2 | Betasil | 7.19 | ESI Neg | 190.0 | 188.0 | 10 | -15 | -10 | -35 | -16 |
| 74 | FLUO3 | 1 | 2 | Betasil | 7.26 | ESI Neg | 181.0 | 153.0 | 10 | -15 | -10 | -30 | -16 |
| | FLUO3-$d_9$ | 1 | 2 | Betasil | 7.17 | ESI Neg | 190.0 | 162.0 | 10 | -15 | -10 | -35 | -16 |
| 75 | NAP1 | 1 | 2 | Betasil | 6.69 | ESI Neg | 143.0 | 115.0 | 20 | -15 | -10 | -35 | -16 |
| | NAP1-$d_7$ | 1 | 2 | Betasil | 6.69 | ESI Neg | 150.0 | 122.0 | 20 | -60 | -10 | -38 | -16 |
| 76 | NAP2 | 1 | 2 | Betasil | 6.51 | ESI Neg | 143.0 | 115.0 | 20 | -15 | -10 | -35 | -16 |
| | NAP2-$d_7$ | 1 | 2 | Betasil | 6.51 | ESI Neg | 150.0 | 122.0 | 20 | -60 | -10 | -38 | -16 |
| 77 | PHEN1 | 1 | 2 | Betasil | 8.00 | ESI Neg | 193.0 | 165.0 | 10 | -15 | -10 | -35 | -16 |
| | PHEN1-$d_9$ | 1 | 2 | Betasil | 8.00 | ESI Neg | 202.0 | 174.0 | 10 | -15 | -10 | -35 | -16 |
| 78 | PHEN2 | 1 | 2 | Betasil | 7.68 | ESI Neg | 193.0 | 165.0 | 10 | -15 | -10 | -35 | -16 |
| | PHEN2-$d_9$ | 1 | 2 | Betasil | 7.68 | ESI Neg | 202.0 | 174.0 | 10 | -15 | -10 | -35 | -16 |
| 79 | PHEN3 | 1 | 2 | Betasil | 7.68 | ESI Neg | 193.0 | 165.0 | 10 | -15 | -10 | -35 | -16 |
| | PHEN3-$d_9$ | 1 | 2 | Betasil | 7.68 | ESI Neg | 202.0 | 174.0 | 10 | -15 | -10 | -35 | -16 |
| 80 | PYR1 | 1 | 2 | Betasil | 8.52 | ESI Neg | 217.0 | 189.0 | 10 | -15 | -10 | -50 | -16 |
| | PYR1-$d_9$ | 1 | 2 | Betasil | 8.47 | ESI Neg | 226.1 | 198.0 | 10 | -60 | -10 | -38 | -16 |
| **Psychosocial Stress Biomarkers** | | | | | | | | | | | | | |
| 81 | CORTE | 1 | 1 | Hypersil | 12.3.5 | ESI Pos | 361.1 | 163.1 | 10 | 76 | 10 | 33 | 16 |
| | CORTE -$d_8$ | 1 | 1 | Hypersil | 12.35 | ESI Pos | 369.2 | 168.1 | 10 | 76 | 10 | 35 | 10 |
| 82 | CORTL | 1 | 1 | Hypersil | 12.55 | ESI Pos | 363.2 | 120.9 | 10 | 106 | 10 | 37 | 16 |
| | CORTL -$d_6$ | 1 | 1 | Hypersil | 12.51 | ESI Pos | 369.1 | 124.1 | 10 | 106 | 10 | 33 | 12 |
| **Tobacco Smoke and Metabolites** | | | | | | | | | | | | | |
| 83 | COTT | 1 | 3 | Kinetex | 2.51 | ESI Pos | 177.2 | 80.1 | 10 | 41 | 3 | 33 | 4 |
| | COTT-$d_3$ | 1 | 3 | Kinetex | 2.51 | ESI Pos | 180.2 | 80.2 | 10 | 36 | 3 | 33 | 4 |

| Analyte # | Analyte Code | Extract # | Injection # | LC column | RT (min) | MS ionization mode | Precursor ion (m/z) | Quantifier product ion (m/z) | Dwell time (ms) | DP (V) | EP (V) | CE (eV) | CXP (V) |
|---|---|---|---|---|---|---|---|---|---|---|---|---|---|
| 84 | HCOTT | 1 | 3 | Kinetex | 2.47 | ESI Pos | 193.2 | 80.2 | 10 | 46 | 8 | 35 | 4 |
| | HCOTT-$d_3$ | 1 | 3 | Kinetex | 2.47 | ESI Pos | 196.2 | 79.9 | 10 | 46 | 10 | 38 | 4 |
| 85 | HYPYBUT | 1 | 3 | Kinetex | 2.40 | ESI Pos | 182.1 | 108.0 | 10 | 55 | 6 | 45 | 4 |
| | HYPYBUT -$d_3$ | 1 | 3 | Kinetex | 2.40 | ESI Pos | 185.2 | 109.0 | 10 | 65 | 10 | 45 | 4 |
| 86 | NCOTT | 1 | 3 | Kinetex | 2.44 | ESI Pos | 163.1 | 80.2 | 10 | 65 | 10 | 33 | 4 |
| | NCOTT-$d_4$ | 1 | 3 | Kinetex | 2.44 | ESI Pos | 167.1 | 84.3 | 10 | 70 | 10 | 34 | 4 |
| 87 | NICT | 1 | 3 | Kinetex | 2.48 | ESI Pos | 163.0 | 132.2 | 10 | 35 | 5.5 | 21 | 4 |
| | NICT_$d_4$ | 1 | 3 | Kinetex | 2.48 | ESI Pos | 167.2 | 136.1 | 10 | 35 | 6 | 25 | 4 |
| 88 | NNICT | 1 | 3 | Kinetex | 2.32 | ESI Pos | 149.0 | 117.2 | 10 | 30 | 10 | 35 | 4 |
| | NNICT-$d_4$ | 1 | 3 | Kinetex | 2.30 | ESI Pos | 153.0 | 121.2 | 10 | 30 | 10 | 35 | 4 |
| 89 | NOXT | 1 | 3 | Kinetex | 2.50 | ESI Pos | 179.2 | 130.1 | 10 | 55 | 10 | 30 | 4 |
| | NOXT_$d_3$ | 1 | 3 | Kinetex | 2.50 | ESI Pos | 182.1 | 130.3 | 10 | 55 | 10 | 31 | 4 |
| **Volatile Organic Compound (VOC) Metabolites** | | | | | | | | | | | | | |
| 90 | AAMA | 1 | 2 | Betasil | 1.45 | ESI Neg | 233.0 | 104.0 | 10 | -5 | -10 | -20 | -15 |
| | AAMA-$d_4$ | 1 | 2 | Betasil | 1.45 | ESI Neg | 237.1 | 108.0 | 10 | -55 | -10 | -20 | -11 |
| 91 | BMA | 1 | 2 | Betasil | 5.65 | ESI Neg | 251.9 | 123.1 | 10 | -50 | -10 | -22 | -11 |
| | SPMA-$d_5$ | 1 | 2 | Betasil | 5.56 | ESI Neg | 242.9 | 114.0 | 10 | -50 | -10 | -32 | -11 |
| 92 | CEMA | 1 | 2 | Betasil | 1.47 | ESI Neg | 233.9 | 161.9 | 10 | -45 | -10 | -14 | -9 |
| | CEMA-$d_3$ | 1 | 2 | Betasil | 1.47 | ESI Neg | 237.0 | 104.9 | 10 | -55 | -10 | -20 | -11 |
| 93 | CYMA | 1 | 2 | Betasil | 1.47 | ESI Neg | 214.9 | 86.0 | 10 | -45 | -10 | -20 | -9 |
| | CEMA-$d_3$ | 1 | 2 | Betasil | 1.47 | ESI Neg | 237.0 | 104.9 | 10 | -55 | -10 | -20 | -11 |
| 94 | DHBMA | 1 | 2 | Betasil | 1.44 | ESI Neg | 250.0 | 121.0 | 10 | -60 | -10 | -20 | -17 |
| | HEMA2-$d_3$ | 1 | 2 | Betasil | 1.44 | ESI Neg | 210.0 | 80.9 | 10 | -75 | -10 | -18 | -11 |
| 95 | HEMA2 | 1 | 2 | Betasil | 1.44 | ESI Neg | 206.0 | 77.0 | 10 | -39 | -10 | -16 | -24 |
| | HEMA2-$d_3$ | 1 | 2 | Betasil | 1.44 | ESI Neg | 210.0 | 80.9 | 10 | -75 | -10 | -18 | -11 |
| 96 | HPMA | 1 | 1 | Hypersil | 6.70 | ESI Neg | 219.9 | 90.9 | 10 | -20 | -10 | -10 | -18 |
| | HPMA-$d_6$ | 1 | 1 | Hypersil | 6.70 | ESI Neg | 226.0 | 97.0 | 10 | -35 | -10 | -10 | -15 |
| 97 | HPMA2 | 1 | 1 | Hypersil | 6.84 | ESI Neg | 219.9 | 91.0 | 10 | -85 | -10 | -10 | -16 |
| | HPMA2-$d_3$ | 1 | 1 | Hypersil | 6.84 | ESI Neg | 223.0 | 91.0 | 10 | -40 | -10 | -10 | -20 |
| 98 | MHA2 | 1 | 1 | Hypersil | 8.99 | ESI Pos | 194.0 | 119.0 | 10 | 50 | 10 | 19 | 17 |
| | THX-$d_3$ | 1 | 1 | Hypersil | 9.14 | ESI Pos | 294.9 | 214.1 | 10 | 41 | 10 | 19 | 12 |
| 99 | MHA34 | 1 | 1 | Hypersil | 9.61 | ESI Pos | 194.0 | 119.0 | 10 | 50 | 10 | 19 | 7 |
| | THX-$d_3$ | 1 | 1 | Hypersil | 9.14 | ESI Pos | 294.9 | 214.1 | 10 | 41 | 10 | 19 | 12 |
| 100 | SPMA | 1 | 2 | Betasil | 5.56 | ESI Neg | 238.0 | 109.0 | 10 | -55 | -10 | -32 | -11 |
| | SPMA-$d_5$ | 1 | 2 | Betasil | 5.56 | ESI Neg | 242.9 | 114.0 | 10 | -50 | -10 | -32 | -11 |

**Supplementary Methods Table S4**. Limits of detection for the analytes studied.

| # | Chemical Group and Full Analyte Name | Analyte Code | LOD (ng/mL) |
|---|---|---|---|
| | **Flame Retardant Metabolites** | | |
| 1 | Bis(2-chloroethyl) phosphate | BCETP | 0.3 |
| 2 | Bis(1,3-dichloro-2-propyl) phosphate | BDCPP | 0.5 |
| 3 | Dibutyl phosphate | DBUP | 1.0 |
| 4 | Diphenyl phosphate | DPHP | 0.2 |
| | **Oxidative Stress Markers** | | |
| 5 | 8-Iso Prostaglandin F2α | F2A8IP | 0.1 |
| 6 | 4-Hydroxy-2-nonenal mercapturic acid | HNEMA | 0.1 |
| | **Personal Care and Consumer Product Chemicals and Metabolites** | | |
| 7 | Triclocarban | TCC | 0.1 |
| 8 | Triclosan | TCS | 1.7 |
| 9 | Bisphenol A | BPA | 0.4 |
| 10 | Bisphenol S | BPS | 0.1 |
| 11 | 4,4'-Cyclo-hexylidenebisphenol | BPZ | 0.05 |
| 12 | Butyl paraben | BUPB | 0.1 |
| 13 | Ethyl paraben | ETPB | 0.2 |
| 14 | Methyl Paraben | MEPB | 0.1 |
| 15 | Propyl paraben | PRPB | 0.1 |
| 16 | Benzophenone-1 | BP1 | 0.1 |
| 17 | Benzophenone-3 | BP3 | 0.4 |
| | **Pesticides and Metabolites** | | |
| 18 | 5-Hydroxythiabendazole | OHTBZ | 0.05 |
| 19 | Pentachlorophenol | PCP | 0.1 |
| 20 | Cis-1,2,3,6-Tetrahydrophthalimide | THPI | 0.1 |
| 21 | 2,4-Dichlorophenoxyacetic acid | D24 | 0.1 |
| 22 | 3-(Diethylcarbamoyl) benzoic acid | DCBA | 0.1 |
| 23 | 3-(Ethylcarbamoyl) benzoic acid | ECBA | 0.1 |
| 24 | Acetamiprid | ACE | 0.1 |
| 25 | 6-Chloronicotinic acid | CINA6 | 0.1 |
| 26 | Clothianidin | CLO | 0.1 |
| 27 | Imidacloprid | IMI | 0.1 |
| 28 | N-Desmethyl-acetamiprid | NDMA | 0.1 |
| 29 | N-Desmethyl thiamethoxam | NDMT | 0.1 |
| 30 | Nitenpyram | NIT | 0.4 |
| 31 | Imidacloprid-olefin | OFIMI | 0.1 |
| 32 | 5-Hydroxyimidacloprid | OHIMI | 0.1 |
| 33 | Diethyldithiophosphate | DEDP | 0.2 |
| 34 | Diethylphosphate | DEP | 0.1 |
| 35 | Diethylthiophosphate | DETP | 0.8 |

| | | | |
|---|---|---|---|
| 36 | Dimethyldithiophosphate | DMDP | 0.1 |
| 37 | Dimethylphosphate | DMP | 0.8 |
| 38 | Dimethylthiophosphate | DMTP | 0.1 |
| 39 | 2-Isopropyl-4-methyl-6-hydroxypyrimidine | IMPY | 0.1 |
| 40 | Malathion dicarboxylic acid | MDA | 0.1 |
| 41 | 4-Nitrophenol | PNP | 0.1 |
| 42 | 3,5,6-Trichloro-2-pyridinol | TCP | 0.1 |
| 43 | Cis-3-(2,2-dichlorovinyl)-2,2-dimethyl-cyclopropane-1-carboxylic acid | CDCCA | 0.1 |
| 44 | 3-Phenoxybenzoic acid | PBA | 0.1 |
| 45 | Trans-3-(2,2-dichlorovinyl)-2,2-dimethyl-cyclopropane-1-carboxylic acid | TDCCA | 0.1 |
| | **Phthalate and Phthalate Alternative Metabolites** | | |
| 46 | Mono-benzyl phthalate | MBZP | 0.1 |
| 47 | Mono-carboxy isononyl phthalate | MCINP | 0.1 |
| 48 | Mono-carboxy isooctyl phthalate | MCIOP | 0.1 |
| 49 | Cyclohexane-1,2-dicarboxylic acid mono carboxyisooctyl ester | MCOCH | 0.05 |
| 50 | Mono (3-carboxypropyl) phthalate | MCPP | 0.1 |
| 51 | Mono-(2-ethyl-5-carboxypentyl) phthalate | MECPP | 0.1 |
| 52 | Mono-2-ethyl-5- carboxypentyl terephthalate | MECPTP | 0.1 |
| 53 | Mono (2-ethyl-5-hydroxyhexyl) phthalate | MEHHP | 0.1 |
| 54 | Mono-2-ethyl-5-hydroxyhexyl terephthalate | MEHHTP | 0.1 |
| 55 | Mono 2-ethyl hexyl phthalate | MEHP | 0.1 |
| 56 | Mono-(2-ethylhexyl) terephthalate | MEHTP | 0.1 |
| 57 | Mono (2-ethyl-5-oxohexyl) phthalate | MEOHP | 0.1 |
| 58 | Mono-2-ethyl-5-oxohexylterephthalate | MEOHTP | 0.1 |
| 59 | Monoethyl phthalate | MEP | 0.1 |
| 60 | Cyclohexane-1,2-dicarboxylic acid mono hydroxyisononyl ester | MHNCH | 0.1 |
| 61 | Mono-isobutyl phthalate | MIBP | 0.2 |
| 62 | Mono-methyl phthalate | MMP | 0.2 |
| 63 | Mono-n-butylphthalate | MNBP | 0.1 |
| 64 | Cyclohexane-1,2-dicarboxylic acid-mono(oxo-isononyl) ester | MONCH | 0.1 |
| 65 | Monooxoisononyl phthalate | MONP | 0.1 |
| 66 | Mono-2-(propyl-6-carboxy-hexyl)-phthalate | MPCHP | 0.1 |
| 67 | Mono-2-(propyl-6-hydroxy-heptyl)-phthalate | MPHHP | 0.1 |
| 68 | Mono-2-(propyl-6-oxoheptyl)-phthalate | MPOHP | 0.1 |
| | **Phytoestrogens and Metabolites** | | |
| 69 | Daidzein | DAZ | 0.7 |
| 70 | Equol | EQU | 0.5 |
| 71 | Enterolactone | ETL | 0.1 |
| 72 | Genistein | GNS | 0.5 |
| | **Polycyclic Aromatic Hydrocarbon Metabolites** | | |
| 73 | 2-Hydroxyfluorene | FLUO2 | 0.1 |

| | | | |
|---|---|---|---|
| 74 | 3-Hydroxyfluorene | FLUO3 | 0.1 |
| 75 | 1-Hydroxynaphthalene | NAP1 | 0.1 |
| 76 | 2-Hydroxynaphthalene | NAP2 | 0.4 |
| 77 | 1-Hydroxyphenanthrene | PHEN1 | 0.1 |
| 78 | 2-Hydroxyphenanthrene | PHEN2 | 0.1 |
| 79 | 3-Hydroxyphenanthrene | PHEN3 | 0.05 |
| 80 | 1-Hydroxypyrene | PYR1 | 0.1 |
| | **Psychosocial Stress Markers** | | |
| 81 | Cortisone | CORTE | 0.1 |
| 82 | Cortisol | CORTL | 0.1 |
| | **Tobacco Metabolites** | | |
| 83 | Cotinine (total) | COTT | 0.04 |
| 84 | 3'-Hydroxycotinine (total) | HCOTT | 0.1 |
| 85 | 4-Hydroxy-4-(3-pyridyl)- butanoic acid (total) | HYPYBUT | 0.2 |
| 86 | Norcotinine (total) | NCOTT | 0.05 |
| 87 | Nicotine (total) | NICT | 0.2 |
| 88 | Nornicotine (total) | NNICT | 1.0 |
| 89 | Nicotine 1'-Oxide (total) | NOXT | 0.05 |
| | **Volatile Organic Compound (VOC) Metabolites** | | |
| 90 | N-Acetyl-S-(2-carbamoylethyl)-L-cysteine | AAMA | 0.3 |
| 91 | N-Acetyl-S-(benzyl)-L-cysteine | BMA | 0.1 |
| 92 | N-Acetyl-S- (2-carboxyethyl)-L-cysteine | CEMA | 0.6 |
| 93 | N-Acetyl-S-(2-cyanoethyl)-L-cysteine | CYMA | 0.1 |
| 94 | N-Acetyl-S- (3,4-dihydroxybutyl)-L-cysteine | DHBMA | 0.1 |
| 95 | N-Acetyl-S- (2-hydroxyethyl)-L-cysteine | HEMA2 | 0.3 |
| 96 | N-Acetyl-S- (3-hydroxypropyl)-L-cysteine | HPMA | 0.4 |
| 97 | N-Acetyl-S-(2-hydroxypropyl)-L-cysteine | HPMA2 | 0.1 |
| 98 | 2-Methylhippuric acid | MHA2 | 0.1 |
| 99 | 3-Methylhippuric acid + 4-Methylhippuric acid | MHA34 | 0.1 |
| 100 | N-acetyl-S-phenyl-L-cysteine | SPMA | 0.1 |

**Supplementary Methods Table S5**. Summary of analyte concentrations in the urine QC pools, showcasing results from three replicate analyses of spiked samples at two concentration levels (1 and 10 ng/mL) across five analytical batches.

| Pesticide Class | Analyte Code | Labeled Analyte Code | QC urine pool, Mean conc.*, ng/mL | 1 ng/mL spike in QC urine pool (n = 15, 3 replicates × 5 batches) | | | | 10 ng/mL spike in QC urine pool (n = 15, 3 replicates × 5 batches) | | | |
|---|---|---|---|---|---|---|---|---|---|---|---|
| | | | | EE % ± SD | RE % | RSD % | CV % | EE % ± SD | RE % | RSD % | CV % |
| **Organophosphorus Insecticides: Dialkyl phosphates (generic metabolites)** | | | | | | | | | | | |
| Dimethylphosphate | DMP | DMP-$d_6$ | 1.62 | 109 ± 17 | 9 | 8 | 16 | 93 ± 16 | -7 | 8 | 18 |
| Dimethylthiophosphate | DMTP | DMTP-$d_6$ | 2.90 | 104 ± 3 | 4 | 4 | 2 | 103 ± 10 | 3 | 3 | 10 |
| Dimethyldithiophosphate | DMDP | DMDP-$d_6$ | 0.30 | 99 ± 12 | -1 | 1 | 12 | 105 ± 12 | 5 | 5 | 12 |
| Diethylphosphate | DEP | DEP-$d_{10}$ | 2.13 | 92 ± 4 | -9 | 9 | 4 | 80 ± 7 | -20 | 23 | 9 |
| Diethylthiophosphate | DETP | DETP-$d_{10}$ | 0.25 | 99 ± 9 | 0 | 0 | 10 | 84 ± 8 | -16 | 17 | 10 |
| Diethyldithiophosphate | DEDP | DEDP-$d_{10}$ | 0.12 | 92 ± 19 | -7 | 8 | 21 | 85 ± 9 | -15 | 17 | 11 |
| **Organophosphorus insecticides: Specific metabolites** | | | | | | | | | | | |
| 4-nitrophenol | PNP | PNP-$^{13}C_6$ | 0.70 | 102 ± 7 | 2 | 2 | 6 | 101 ± 7 | 1 | 1 | 7 |
| 3,5,6-trichloro-2-pyridinol | TCP | TCP-$^{13}C_3$ | 0.72 | 111 ± 6 | 10 | 10 | 5 | 114 ± 5 | 14 | 13 | 4 |
| 2-[dimethoxyphosphorothioyl) sulfanyl] succinic acid | MDA | MDA-$d_4$ | 0.16 | 101 ± 5 | 1 | 1 | 5 | 90 ± 8 | -10 | 10 | 9 |
| 2-isopropyl-4-methyl-pyrimidinol | IMPY | IMPY-$^{13}C_4$ | 0.05 | 99 ± 6 | -1 | 2 | 6 | 105 ± 7 | 5 | 5 | 7 |
| **Pyrethroid insecticides** | | | | | | | | | | | |
| trans-dichlorovinyl-dimethylcyclopropane carboxylic acid | TDCCA | TDCCA-$^{13}C_2$ | 2.47 | 107 ± 14 | 7 | 7 | 13 | 94 ± 8 | -6 | 6 | 8 |
| cis-dichlorovinyl-dimethylcyclopropane carboxylic acid | CDCCA | CDCCA-$^{13}C_2$ | 1.12 | 103 ± 8 | 4 | 4 | 7 | 106 ± 12 | 6 | 6 | 11 |
| 3-phenoxybenzoic Acid | 3PBA | 3PBA-$^{13}C_6$ | 1.82 | 102 ± 3 | 2 | 2 | 3 | 90 ± 8 | -10 | 10 | 9 |
| **Fungicides and metabolites** | | | | | | | | | | | |
| Pentachlorophenol | PCP | PCP-$^{13}C_6$ | 0.20 | 95 ± 3 | -5 | 6 | 3 | 106 ± 8 | 6 | 6 | 8 |
| cis-1,2,3,6-tetrahydrophthalimide | THPI | NDMA-$^{13}C_3$ | 19.40 | 98 ± 10 | 0 | 0 | 10 | 77 ± 15 | -20 | 22 | 19 |
| Hydroxy tebuconazole | OH-TBZ | IMZ-d4 | 0.23 | 107 ± 12 | 7 | 7 | 11 | 103 ± 17 | 3 | 3 | 16 |
| **Neonicotinoid insecticides** | | | | | | | | | | | |
| 6-chloronicotinic Acid | CINA6 | CINA6-1$^{3}C_6$ | 0.53 | 92 ± 10 | -8 | 8 | 11 | 76 ± 3 | -24 | 27 | 4 |
| Acetamiprid | ACE | ACE-$d_3$ | 0.06 | 103 ± 9 | 3 | 3 | 9 | 97 ± 4 | -3 | 3 | 4 |
| N-desmethyl-acetamiprid | NDMA | NDMA-$^{13}C_3$ | 1.91 | 100 ± 10 | -1 | 1 | 10 | 89 ± 4 | -11 | 12 | 4 |
| Imidacloprid | IMI | IMI-$d_4$ | 0.46 | 98 ± 4 | -2 | 2 | 4 | 93 ± 3 | -7 | 7 | 3 |
| 5-Hydroxyimidacloprid | OHIMI | OHIMI-$^{13}C^{15}N^{15}N$ | 0.55 | 93 ± 8 | -7 | 7 | 8 | 88 ± 4 | -13 | 13 | 4 |
| Clothianidin | CLO | CLO-$d_3$ | 1.42 | 101 ± 14 | 1 | 1 | 14 | 91 ± 8 | -9 | 10 | 9 |
| Nitenpyram | NIT | NIT-d3 | 0.05 | 113 ± 6 | 13 | 12 | 5 | 93 ± 7 | -7 | 8 | 7 |
| Imidacloprid-olefin | OFIMID | OFIMID-$^{15}N^{13}C^{15}N$ | 11.16 | 93 ± 12 | -8 | 9 | 13 | 68 ± 4 | -32 | 38 | 6 |
| N-desmethyl thiamethoxam | NDMT | IMZ-$d_4$ | 2.03 | 111 ± 10 | 11 | 10 | 9 | 105 ± 17 | 5 | 5 | 16 |
| **Herbicides and metabolites** | | | | | | | | | | | |
| 2,4-dichlorophenoxyacetic acid | D24 | D24-$^{13}C_6$ | 0.41 | 110 ± 4 | 10 | 10 | 4 | 107 ± 4 | 7 | 7 | 3 |
| **Insect repellents and metabolites** | | | | | | | | | | | |

| Pesticide Class | Analyte Code | Labeled Analyte Code | QC urine pool, Mean conc.*, ng/mL | 1 ng/mL spike in QC urine pool (n = 15, 3 replicates × 5 batches) | | | | 10 ng/mL spike in QC urine pool (n = 15, 3 replicates × 5 batches) | | | |
|---|---|---|---|---|---|---|---|---|---|---|---|
| | | | | EE % ± SD | RE % | RSD % | CV % | EE % ± SD | RE % | RSD % | CV % |
| 3-(diethylcarbamoyl) benzoic acid | DCBA | DCBA-$d_{10}$ | 18.45 | 101 ± 2 | 1 | 1 | 2 | 89 ± 4 | -11 | 11 | 4 |
| 3-(ethylcarbamoyl) benzoic acid | ECBA | ECBA-$d_5$ | 11.81 | 98 ± 9 | -3 | 4 | 10 | 88 ± 14 | -13 | 13 | 16 |
| **Volatile Organic Compounds** | | | | | | | | | | | |
| N-Acetyl-S-(2-carbamoylethyl)-L-cysteine | AAMA | AAMA-$d_4$ | 52.19 | 100 ± 6 | -1 | 1 | 6 | 76 ± 1 | -25 | 29 | 14 |
| N-Acetyl-S- (2-hydroxyethyl)-L-cysteine | HEMA | HEMA-$d_3$ | 2.21 | 99 ± 21 | -1 | 1 | 22 | 83 ± 11 | -16 | 18 | 14 |
| N-Acetyl-S- (3,4-dihydroxybutyl)-L-cysteine | DHBMA | HEMA-$d_3$ | 34.40 | 96 ± 9 | -4 | 4 | 9 | 104 ± 20 | 1 | 1 | 19 |
| N-Acetyl-S-(2-cyanoethyl)-L-cysteine | CEMA | CEMA-$d_3$ | 16.15 | 89 ± 17 | -6 | 6 | 19 | 87 ± 17 | -11 | 12 | 20 |
| N-Acetyl-S- (3-hydroxypropyl)-L-cysteine | 3-HPMA | 3-HPMA-$d_6$ | 347.13 | 102 ± 9 | 2 | 2 | 9 | 83 ± 10 | -18 | 20 | 13 |
| N-Acetyl-S-(2-hydroxypropyl)-L-cysteine | 2-HPMA | 2-HPMA-$d_3$ | 18.43 | 117 ± 11 | 14 | 13 | 9 | 108 ± 26 | 0 | 0 | 24 |
| N-acetyl-S-(phenyl)-L-cysteine | SPMA | SPMA-$d_5$ | 0.15 | 107 ± 13 | 7 | 7 | 12 | 88 ± 14 | -12 | 12 | 15 |
| N-Acetyl-S-(benzyl)-L-Cysteine | BMA | BMA-$d_5$ | 4.71 | 106 ± 4 | 6 | 6 | 4 | 105 ± 7 | 5 | 5 | 7 |
| 2-methylhippuric acid | 2-MHA | 2-MHA-$d_7$ | 23.90 | 97 ± 5 | -4 | 4 | 5 | 74 ± 3 | -26 | 30 | 4 |
| 3-methylhippuric acid | 3-MHA | 3-MHA-$d_7$ | 768.59 | 97 ± 6 | -8 | 8 | 6 | 103 ± 1 | 4 | 4 | 1 |
| 4-methylhippuric acid | 4-MHA | 4-MHA-$d_7$ | 791.01 | 104 ± 7 | -1 | 1 | 6 | 105 ± 10 | 5 | 5 | 10 |
| N-Acetyl-S-(2-cyanoethyl)-L-cysteine | CYMA | CYMA-$d_3$ | 8.64 | 103 ± 4 | 2 | 2 | 4 | 117 ± 3 | 17 | 16 | 3 |
| **OP flame retardants** | | | | | | | | | | | |
| Diphenyl Phosphate | DPHP | DPHP-$d_{10}$ | 0.67 | 99 ± 9 | -1 | 1 | 9 | 85 ± 8 | -15 | 16 | 10 |
| Dibutyl phosphate | DBUP | DBUP-$d_{18}$ | 0.39 | 111 ± 5 | 11 | 11 | 4 | 99 ± 5 | -1 | 1 | 5 |
| Bis(1,3-Dichloro-2-Propyl) Phosphate | BDCPP | BDCPP-$d_{10}$ | 0.70 | 115 ± 7 | 15 | 14 | 6 | 117 ± 7 | 17 | 16 | 6 |
| Bis(2-Chloroethyl) Phosphate | BCETP | BCETP-$d_8$ | 0.20 | 94 ± 17 | -5 | 5 | 18 | 101 ± 20 | 1 | 1 | 20 |
| **Parabens** | | | | | | | | | | | |
| Butyl Paraben | BUPB | BUPB-$^{13}C_6$ | 0.24 | 108 ± 6 | 8 | 8 | 6 | 106 ± 9 | 6 | 6 | 9 |
| Ethyl Paraben | ETPB | ETPB-$^{13}C_6$ | 6.94 | 96 ± 4 | -4 | 4 | 4 | 89 ± 3 | -11 | 11 | 3 |
| Methyl Paraben | MEPB | MEPB-$^{13}C_6$ | 36.73 | 100 ± 2 | 0 | 0 | 2 | 97 ± 4 | -3 | 3 | 4 |
| Propyl Paraben | PRPB | PRPB-$^{13}C_6$ | 5.90 | 97 ± 5 | -3 | 3 | 5 | 91 ± 1 | -9 | 10 | 1 |
| **PAHs** | | | | | | | | | | | |
| 2-Hydroxynaphthalene | 2-NAP | 2-NAP-$d_7$ | 2.58 | 92 ± 6 | -8 | 9 | 7 | 82 ± 3 | -18 | 20 | 4 |
| 1-Hydroxynaphthalene | 1-NAP | 1-NAP-$d_7$ | 0.49 | 94 ± 14 | -5 | 6 | 15 | 101 ± 17 | 1 | 1 | 17 |
| 1-Hydroxypyrene | 1-PYR | 1-PYR-$d_9$ | 0.27 | 84 ± 8 | -16 | 17 | 9 | 131 ± 5 | 31 | 27 | 4 |
| 2-Hydroxyfluorene | 2-OH FLUO | 2-OH FLUO-$d_9$ | 0.17 | 105 ± 8 | 5 | 5 | 7 | 110 ± 13 | 10 | 10 | 12 |
| 3-Hydroxyfluorene | 3-OH FLUO | 3-OH FLUO-$d_9$ | 0.17 | 144 ± 4 | 14 | 13 | 4 | 121 ± 5 | 21 | 19 | 4 |
| 3-Hydroxyphenanthrene | 3-OH PHEN | 3-OH PHEN-$d_9$ | 0.13 | 98 ± 6 | -2 | 2 | 6 | 119 ± 10 | 19 | 17 | 8 |
| 2-Hydroxyphenanthrene | 2-OH PHEN | 2-OH PHEN-$d_9$ | 0.05 | 88 ± 12 | -12 | 13 | 13 | 108 ± 12 | 8 | 8 | 12 |
| 1-Hydroxyphenanthrene | 1-OH PHEN | 1-OH PHEN-$d_9$ | 0.24 | 89 ± 8 | -11 | 11 | 9 | 117 ± 10 | 17 | 15 | 9 |
| **Environmental Phenols** | | | | | | | | | | | |
| Bisphenol A | BPA | BPA-$^{13}C_{12}$ | 1.84 | 99 ± 23 | -7 | 7 | 23 | 86 ± 15 | -13 | 14 | 18 |
| Bisphenol S | BPS | BPS-$^{13}C_{12}$ | 1.56 | 92 ± 4 | -8 | 8 | 5 | 82 ± 5 | -18 | 20 | 6 |
| Bisphenol Z | BPZ | BPZ-d6 | 0.01 | 92 ± 8 | -8 | 9 | 9 | 105 ± 15 | 5 | 5 | 14 |
| Triclosan | TCS | TCS-$^{13}C_{12}$ | 331.85 | 110 ± 10 | 9 | 9 | 9 | 102 ± 9 | 1 | 1 | 9 |
| Triclocarban | TCC | TCC-$^{13}C_6$ | 0.53 | 74 ± 9 | -26 | 29 | 11 | 121 ± 13 | 21 | 19 | 11 |
| Benzophenone-1 | BP1 | BP1-d5 | 11.0 | 96 ± 3 | -4 | 4 | 3 | 84 ± 6 | -16 | 18 | 7 |

| Pesticide Class | Analyte Code | Labeled Analyte Code | QC urine pool, Mean conc.*, ng/mL | 1 ng/mL spike in QC urine pool (n = 15, 3 replicates × 5 batches) | | | | 10 ng/mL spike in QC urine pool (n = 15, 3 replicates × 5 batches) | | | |
|---|---|---|---|---|---|---|---|---|---|---|---|
| | | | | EE % ± SD | RE % | RSD % | CV % | EE % ± SD | RE % | RSD % | CV % |
| Benzophenone-3 | BP3 | BP3-$^{13}C_6$ | 43.51 | 100 ± 2 | 0 | 0 | 2 | 87 ± 7 | -12 | 12 | 9 |
| **Tobacco Metabolites** | | | | | | | | | | | |
| Nicotine (total) | NIC | NIC-$d_4$ | 17.57 | 97 ± 8 | -4 | 4 | 8 | 92 ± 8 | -8 | 9 | 9 |
| Cotinine (total) | COT | COT-$d_3$ | 53.69 | 100 ± 2 | 0 | 0 | 2 | 94 ± 2 | -6 | 6 | 3 |
| Hydroxycotinine (total) | HCOT | HCOT-$d_3$ | 42.77 | 100 ± 2 | 0 | 0 | 2 | 99 ± 5 | -1 | 1 | 5 |
| 4-hydroxy-4-(3-pyridyl)- butanoic acid (total) | HyPyBut | HyPyBut-$d_3$ | 12.17 | 98 ± 6 | -2 | 2 | 6 | 94 ± 6 | -6 | 6 | 6 |
| Norcotinine | NCOT | NCOT-$d_4$ | 17.57 | 96 ± 3 | -4 | 4 | 3 | 97 ± 8 | -3 | 3 | 8 |
| Nornicotine | NNIC | NNIC-$d_4$ | 4.12 | 103 ± 9 | 2 | 2 | 8 | 121 ± 3 | 21 | 19 | 3 |
| Nicotine N-oxide | NOX | NOX-$d_3$ | 16.09 | 99 ± 4 | -2 | 2 | 4 | 88 ± 4 | -12 | 13 | 5 |
| **Psychosocial stress biomarkers** | | | | | | | | | | | |
| Cortisone | Cortisone | Cortisone-$d_8$ | 67.56 | 100 ± 6 | 0 | 0 | 6 | 93 ± 4 | -7 | 8 | 4 |
| Cortisol | Cortisol | Cortisol-$d_6$ | 28.23 | 97 ± 6 | -4 | 4 | 6 | 91 ± 8 | -9 | 9 | 9 |
| **Lipid peroxidation products** | | | | | | | | | | | |
| N-acetyl-S-(tetrahydro-5-hydroxy-2-pentyl-3-furanyl)-L-cysteine | HNE-MA | HNE-MA-$d_3$ | 113.48 | 100 ± 4 | 0 | 0 | 4 | 91 ± 3 | -9 | 9 | 4 |
| 8-Iso Prostaglandin F2A / 8-iso-PGF2a | 8-ISO-PGF2a | 8-ISO-PGF2a-$d_4$ | 0.13 | 95 ± 9 | -5 | 6 | 10 | 103 ± 8 | 3 | 3 | 8 |
| **Phytoestrogens and metabolites** | | | | | | | | | | | |
| Daidzein | Daidzein | Diadzein-$d_4$ | 209.40 | 98 ± 5 | -3 | 3 | 5 | 96 ± 5 | -4 | 5 | 5 |
| Enterolactone | Enterolactone | Enterolactone-$^{13}C_3$ | 531.51 | 97 ± 4 | -3 | 3 | 4 | 96 ± 4 | -4 | 4 | 4 |
| Equol | S-Equol | S-Equol-$d_4$ | 21.47 | 101 ± 2 | 1 | 1 | 2 | 92 ± 9 | -7 | 8 | 10 |
| Genistein | Genistein | Genistein-$d_4$ | 114.55 | 99 ± 2 | -1 | 1 | 2 | 94 ± 5 | -6 | 6 | 5 |
| **Phthalates** | | | | | | | | | | | |
| Mono-2-ethyl-5-carboxypentyl phthalate | MECPP | MECPP-$^{13}C_4$ | 4.39 | 96 ± 2 | -4 | 4 | 2 | 88 ± 10 | -12 | 12 | 11 |
| Mono-2-ethyl-5-oxohexyl phthalate | MEOHP | MEOHP-$^{13}C_4$ | 2.28 | 96 ± 4 | -4 | 4 | 4 | 86 ± 9 | -14 | 15 | 10 |
| Mono-isobutyl phthalate | MIBP | MIBP-$^{13}C_2{}^{13}C_2$ | 3.71 | 110 ± 15 | 10 | 9 | 14 | 96 ± 5 | -5 | 5 | 6 |
| Mono-n-butylphthalate | MNBP | MNBP-$^{13}C_2{}^{13}C_2$ | 8.62 | 95 ± 10 | -6 | 6 | 10 | 87 ± 10 | -13 | 13 | 12 |
| Mono-2-ethyl-5-hydroxyhexyl phthalate | MEHHP | MEHHP-$^{13}C_4$ | 4.47 | 96 ± 8 | -4 | 4 | 8 | 97 ± 6 | -3 | 3 | 6 |
| Mono-benzyl phthalate | MBZP | MBZP-$^{13}C_2{}^{13}C_2$ | 1.10 | 94 ± 3 | -6 | 6 | 4 | 77 ± 4 | -23 | 26 | 5 |
| Mono-carboxyoctyl phthalate isomers | MCOP | MCOP-$^{13-}C_2{}^{13}C_2$ | 3.55 | 100 ± 4 | 0 | 0 | 4 | 88 ± 8 | -12 | 13 | 9 |
| Mono-oxo-iso-nonyl phthalate | MONP | MONP-$^{13}C_2{}^{13}C_2$ | 1.80 | 102 ± 4 | 2 | 2 | 4 | 95 ± 5 | -5 | 6 | 5 |
| Mono-(2-ethyl-5-carboxypentyl) terephthalate | MECPTP | MECPTP-$d_4$ | 171.55 | 100 ± 1 | 0 | 0 | 1 | 96 ± 2 | -4 | 4 | 3 |
| Mono-(2-ethyl-5-hydroxyhexyl) terephthalate | MEHHTP | MEHHTP-$d_4$ | 15.45 | 101 ± 6 | 1 | 1 | 6 | 89 ± 6 | -11 | 12 | 6 |
| Mono-(2-ethyl-5-oxo-hexyl) terephthalate | MEOHTP | MEHHTP-$d_4$ | 4.62 | 98 ± 4 | -2 | 2 | 4 | 78 ± 5 | -22 | 25 | 6 |
| Mono-carboxynonyl phthalate isomers | MCNP | MCNP-$^{13}C_2{}^{13}C_2$ | 1.02 | 113 ± 10 | 13 | 12 | 9 | 105 ± 16 | 5 | 5 | 16 |
| Mono (2-propyl-6-carboxyhexyl) phthalate | MPCHP | MPCHP-$^{13}C_2{}^{13}C_2$ | 0.90 | 109 ± 11 | 10 | 10 | 10 | 103 ± 10 | 3 | 3 | 10 |
| Mono (2-propyl-6-oxo-heptyl) phthalate | MPOHP | MPOHP-$^{13}C_2{}^{13}C_2$ | 2.65 | 100 ± 5 | -1 | 1 | 5 | 94 ± 8 | -6 | 6 | 9 |
| Cyclohexane-1,2-dicarboxylate-mono-(7-carboxylate-4-methyl)heptyl ester | MCOCH | MCOCH-$^{13}C_4$ | 2.68 | 106 ± 5 | 6 | 6 | 5 | 105 ± 12 | 5 | 5 | 11 |
| Mono (2-propyl-6-hydroxyheptyl) phthalate | MPHHP | MPHHP-$^{13}C_2{}^{13}C_2$ | 2.62 | 98 ± 8 | -4 | 4 | 8 | 100 ± 5 | 0 | 0 | 5 |
| Cyclohexane-1,2-dicarboxylate-mono-(7-oxo-4-methyl)octyl ester | MONCH | MONCH-$^{13}C_4$ | 5.32 | 97 ± 7 | -3 | 3 | 8 | 98 ± 12 | -2 | 2 | 12 |
| Cyclohexane-1,2-dicarboxylate-mono-(7-hydroxy-4-methyl)octyl ester | MHNCH | MHNCH-$^{13}C_4$ | 21.62 | 97 ± 3 | -3 | 3 | 3 | 92 ± 5 | -8 | 8 | 6 |
| Mono-2-ethylhexyl phthalate | MEHP | MEHP-$^{13}C_2{}^{13}C_2$ | 1.38 | 96 ± 3 | -4 | 4 | 3 | 87 ± 9 | -13 | 14 | 11 |
| Mono-2-ethylhexyl terephthalate | MEHTP | MEHTP-$^{13}C_6$ | 1.38 | 95 ± 15 | -7 | 7 | 15 | 91 ± 15 | -12 | 13 | 17 |

| Pesticide Class | Analyte Code | Labeled Analyte Code | QC urine pool, Mean conc.*, ng/mL | 1 ng/mL spike in QC urine pool (n = 15, 3 replicates × 5 batches) | | | | 10 ng/mL spike in QC urine pool (n = 15, 3 replicates × 5 batches) | | | |
|---|---|---|---|---|---|---|---|---|---|---|---|
| | | | | EE % ± SD | RE % | RSD % | CV % | EE % ± SD | RE % | RSD % | CV % |
| Mono-(3-carboxypropyl) phthalate | MCPP | MCPP-$^{13}C_2{}^{13}C_2$ | 8.13 | 127 ± 8 | 27 | 24 | 6 | 100 ± 15 | 1 | 1 | 14 |
| Mono-methyl phthalate | MMP | MMP-$^{13}C_2{}^{13}C_2$ | 12.15 | 107 ± 14 | 3 | 3 | 13 | 94 ± 11 | -7 | 7 | 11 |
| Monoethyl phthalate | MEP | MEP-$^{13}C_2{}^{13}C_2$ | 15.48 | 99 ± 2 | -1 | 1 | 2 | 83 ± 6 | -17 | 18 | 7 |

*Average of the instrument readouts were presented for the QC urine pool, despite some < LOD.

## CV Anova - PLS regression models:

**Supplementary Methods Table S6**.

| High BMI Glucocorticoids | SS | DF | MS | F | p | SD |
|---|---|---|---|---|---|---|
| CORTL | | | | | | |
| Total corr. | 53 | 53 | 1 | | | 1 |
| Regression | 16.9559 | 4 | 4.23898 | 5.76267 | 0.000698 | 2.05888 |
| Residual | 36.0441 | 49 | 0.735593 | | | 0.857667 |
| CORTE | | | | | | |
| Total corr. | 53 | 53 | 1 | | | 1 |
| Regression | 30.1316 | 4 | 7.5329 | 16.1407 | 1.7E-08 | 2.74461 |
| Residual | 22.8684 | 49 | 0.466702 | | | 0.683156 |

**Supplementary Methods Table S7**.

| **Low BMI Glucocorticoids** | **SS** | **DF** | **MS** | **F** | **p** | **SD** |
|---|---|---|---|---|---|---|
| CORTL | | | | | | |
| Total corr. | 62 | 62 | 1 | | | 1 |
| Regression | 25.0622 | 6 | 4.17704 | 6.33265 | 3.96E-05 | 2.04378 |
| Residual | 36.9378 | 56 | 0.659603 | | | 0.81216 |
| CORTE | | | | | | |
| Total corr. | 62 | 62 | 1 | | | 1 |
| Regression | 39.3178 | 6 | 6.55297 | 16.1786 | 1.08E-10 | 2.55988 |
| Residual | 22.6822 | 56 | 0.405039 | | | 0.636427 |

**Supplementary Methods Table S8**.

| **High BMI Oxidative Stress** | **SS** | **DF** | **MS** | **F** | **p** | **SD** |
|---|---|---|---|---|---|---|
| F2A8IP | | | | | | |
| Total corr. | 53 | 53 | 1 | | | 1 |
| Regression | 27.9355 | 4 | 6.98388 | 13.6532 | 1.5E-07 | 2.6427 |
| Residual | 25.0645 | 49 | 0.51152 | | | 0.715206 |

| | | | | | | |
|---|---|---|---|---|---|---|
| HNEMA | | | | | | |
| Total corr. | 53 | 53 | 1 | | | 1 |
| Regression | 23.9705 | 4 | 5.99262 | 10.1152 | 4.75E-06 | 2.44798 |
| Residual | 29.0295 | 49 | 0.592439 | | | 0.769701 |

**Supplementary Methods Table S9**.

| **Low BMI** | **SS** | **DF** | **MS** | **F** | **p** | **SD** |
|---|---|---|---|---|---|---|
| F2A8IP | | | | | | |
| Total corr. | 62 | 62 | 1 | | | 1 |
| Regression | 32.4566 | 2 | 16.2283 | 32.9583 | 2.2E-10 | 4.02844 |
| Residual | 29.5434 | 60 | 0.492389 | | | 0.701705 |
| HNEMA | | | | | | |
| Total corr. | 62 | 62 | 1 | | | 1 |
| Regression | 17.4878 | 2 | 8.74389 | 11.7863 | 4.82E-05 | 2.95701 |
| Residual | 44.5122 | 60 | 0.741871 | | | 0.861319 |
