# Supplementary material for: Early Pregnancy Targeted Exposome: Biological Response and Maternal BMI
Source: Toxics. 2026 May 12;14(5):421. doi: 10.3390/toxics14050421 (PMC13211517; doi:10.3390/toxics14050421)
Supplement: Supplementary file 1 [file toxics-14-00421-s001.zip › Supplementary Table S1. Analytes.pdf]

Supplementary Table S1. Comprehensive list of all biomarkers of exposure and effect incorporated in this study, including their acronyms, CAS numbers, and chemical classifications.

CAS #	Code	Analyte Name	Sub-Chemical Group	Chemical Group
81690-92-8	AAMA	N-Acetyl-S-(2-carbamoylethyl)-L-cysteine	NA	Volatile Organic Compound (VOC) Metabolites
135410-20-7	ACE	Acetamiprid	Neonicotinoid insecticides	Pesticides and Metabolites
3040-56-0	BCETP	Bis(2-chloroethyl) phosphate	Organophosphorus flame retardants	Flame Retardant Metabolites
72236-72-7	BDCPP	Bis(1,3-dichloro-2-propyl) phosphate	Organophosphorus flame retardants	Flame Retardant Metabolites
19542-77-9	BMA	N-Acetyl-S-(benzyl)-L-cysteine	NA	Volatile Organic Compound (VOC) Metabolites
131-56-6	BP1	Benzophenone-1	UV filters	Personal Care and Consumer Product Chemicals and Metabolites
131-57-7	BP3	Benzophenone-3	UV filters	Personal Care and Consumer Product Chemicals and Metabolites
80-05-7	BPA	Bisphenol A	Bisphenols	Personal Care and Consumer Product Chemicals and Metabolites
80-09-1	BPS	Bisphenol S	Bisphenols	Personal Care and Consumer Product Chemicals and Metabolites
843-55-0	BPZ	Bisphenol Z	Bisphenols	Personal Care and Consumer Product Chemicals and Metabolites
94-26-8	BUPB	Butyl paraben	Parabens	Personal Care and Consumer Product Chemicals and Metabolites
59042-49-8	CDCCA	Cis-3-(2,2-dichlorovinyl)-2,2-dimethyl-cyclopropane-1-carboxylic acid	Pyrethroid metabolites	Pesticides and Metabolites
51868-61-2	CEMA	N-Acetyl-S- (2-carboxyethyl)-L-cysteine	NA	Volatile Organic Compound (VOC) Metabolites
5326-23-8	CINA6	6-Chloronicotinic acid	Neonicotinoid insecticides	Pesticides and Metabolites
210880-92-5	CLO	Clothianidin	Neonicotinoid insecticides	Pesticides and Metabolites
53-06-5	CORTE	Cortisone	NA	Psychosocial Stress Markers
50-23-7	CORTL	Cortisol	NA	Psychosocial Stress Markers
486-56-6	COTT	Cotinine (total)	NA	Tobacco Metabolites
74514-75-3	CYMA	N-Acetyl-S-(2-cyanoethyl)-L-cysteine	NA	Volatile Organic Compound (VOC) Metabolites
94-75-7	D24	2,4-Dichlorophenoxyacetic acid	Herbicides and metabolites	Pesticides and Metabolites
486-66-8	DAZ	Daidzein	NA	Phytoestrogens and Metabolites
107-66-4	DBUP	Dibutyl phosphate	Organophosphorus flame retardants	Flame Retardant Metabolites

72236-23-8	DCBA	3-(Diethylcarbamoyl) benzoic acid	Insect repellent and metabolites	Pesticides and Metabolites
298-06-6	DEDP	Diethyldithiophosphate	Organophosphorus insecticides: Dialkyl phosphate metabolites	Pesticides and Metabolites
598-02-7	DEP	Diethylphosphate	Organophosphorus insecticides: Dialkyl phosphate metabolites	Pesticides and Metabolites
5871-17-0	DETP	Diethylthiophosphate	Organophosphorus insecticides: Dialkyl phosphate metabolites	Pesticides and Metabolites
144889-50-9	DHBMA	N-Acetyl-S- (3,4-dihydroxybutyl)-L-cysteine	NA	Volatile Organic Compound (VOC) Metabolites
756-80-9	DMDP	Dimethyldithiophosphate	Organophosphorus insecticides: Dialkyl phosphate metabolites	Pesticides and Metabolites
813-78-5	DMP	Dimethylphosphate	Organophosphorus insecticides: Dialkyl phosphate metabolites	Pesticides and Metabolites
59401-04-6	DMTP	Dimethylthiophosphate	Organophosphorus insecticides: Dialkyl phosphate metabolites	Pesticides and Metabolites
838-85-7	DPHP	Diphenyl phosphate	Organophosphorus flame retardants	Flame Retardant Metabolites
126926-33-8	ECBA	3-(Ethylcarbamoyl) benzoic acid	Insect repellent and metabolites	Pesticides and Metabolites
531-95-3	EQU	Equol	NA	Phytoestrogens and Metabolites
78473-71-9	ETL	Enterolactone	NA	Phytoestrogens and Metabolites
120-47-8	ETPB	Ethyl paraben	Parabens	Personal Care and Consumer Product Chemicals and Metabolites
27415-26-5	F2A8IP	8-Iso Prostaglandin F2 α	Lipid peroxidation products	Oxidative Stress Markers
2443-58-5	FLUO2	2-Hydroxyfluorene	NA	Polycyclic Aromatic Hydrocarbon (PAH) Metabolites
6344-67-8	FLUO3	3-Hydroxyfluorene	NA	Polycyclic Aromatic Hydrocarbon (PAH) Metabolites
446-72-0	GNS	Genistein	NA	Phytoestrogens and Metabolites
34834-67-8	HCOTT	3'-Hydroxycotinine (total)	NA	Tobacco Metabolites
15060-26-1	HEMA2	N-Acetyl-S- (2-hydroxyethyl)-L-cysteine	NA	Volatile Organic Compound (VOC) Metabolites
146764-24-1	HNEMA	4-Hydroxy-2-nonenal mercapturic acid	Lipid peroxidation products	Oxidative Stress Markers
23127-40-4	HPMA3	N-Acetyl-S- (3-hydroxypropyl)-L-cysteine	NA	Volatile Organic Compound (VOC) Metabolites
923-43-3	HPMA2	N-Acetyl-S-(2-hydroxypropyl)-L-cysteine	NA	Volatile Organic Compound (VOC) Metabolites

15569-97-8	HYPYBUT	4-Hydroxy-4-(3-pyridyl)-butanoic acid (total)	NA	Tobacco Metabolites
138261-41-3	IMI	Imidacloprid	Neonicotinoid insecticides	Pesticides and Metabolites
2814-20-2	IMPY	2-Isopropyl-4-methyl-6-hydroxypyrimidine	Organophosphorus insecticides: Specific metabolites	Pesticides and Metabolites
105843-36-5	IMZ	Imidaclothiz	Neonicotinoid insecticides	Pesticides and Metabolites
2528-16-7	MBZP	Mono-benzyl phthalate	NA	Phthalate and Phthalate Alternative Metabolites
1373125-93-9	MCINP	Mono-carboxy isononyl phthalate	NA	Phthalate and Phthalate Alternative Metabolites
898544-09-7	MCIOP	Mono-carboxy isooctyl phthalate	NA	Phthalate and Phthalate Alternative Metabolites
1637562-51-6	MCOCH	Cyclohexane-1,2-dicarboxylic acid mono carboxyisooctyl ester	NA	Phthalate and Phthalate Alternative Metabolites
66851-46-5	MCPP	Mono-(3-carboxypropyl) phthalate	NA	Phthalate and Phthalate Alternative Metabolites
1190-28-9	MDA	Malathion dicarboxylic acid	Organophosphorus insecticides: Specific metabolites	Pesticides and Metabolites
40809-41-4	MECPP	Mono-(2-ethyl-5-carboxypentyl) phthalate	NA	Phthalate and Phthalate Alternative Metabolites
1684398-42-2	MECPTP	Mono-2-ethyl-5-carboxypentyl terephthalate	NA	Phthalate and Phthalate Alternative Metabolites
40321-99-1	MEHHP	Mono-(2-ethyl-5-hydroxyhexyl) phthalate	NA	Phthalate and Phthalate Alternative Metabolites
1684398-38-6	MEHHTP	Mono-2-ethyl-5-hydroxyhexyl terephthalate	NA	Phthalate and Phthalate Alternative Metabolites
4376-20-9	MEHP	Mono-2-ethyl hexyl phthalate	NA	Phthalate and Phthalate Alternative Metabolites
155603-50-2	MEHTP	Mono-(2-ethylhexyl) terephthalate	NA	Phthalate and Phthalate Alternative Metabolites
40321-98-0	MEOHP	Mono-(2-ethyl-5-hydroxyhexyl) phthalate	NA	Phthalate and Phthalate Alternative Metabolites
NA	MEOHTP	Mono-2-ethyl-5-oxohexylterephthalate	NA	Phthalate and Phthalate Alternative Metabolites
2306-33-4	MEP	Mono-ethyl phthalate	NA	Phthalate and Phthalate Alternative Metabolites
99-76-3	MEPB	Methyl Paraben	Parabens	Personal Care and Consumer Product Chemicals and Metabolites
42013-20-7	MHA2	2-Methylhippuric acid	NA	Volatile Organic Compound (VOC) Metabolites
NA	MHA34	3-Methylhippuric acid + 4-Methylhippuric acid	NA	Volatile Organic Compound (VOC) Metabolites
1637562-52-7	MHNCH	Cyclohexane-1,2-dicarboxylic acid mono hydroxyisononyl ester	NA	Phthalate and Phthalate Alternative Metabolites
30833-53-5	MIBP	Mono-isobutyl phthalate	NA	Phthalate and Phthalate Alternative Metabolites

4376-18-5	MMP	Mono-methyl phthalate	NA	Phthalate and Phthalate Alternative Metabolites
131-70-4	MNBP	Mono-n-butylphthalate	NA	Phthalate and Phthalate Alternative Metabolites
1588520-62-0	MONCH	Cyclohexane-1,2-dicarboxylic acid-mono(oxo-isononyl) ester	NA	Phthalate and Phthalate Alternative Metabolites
936022-00-3	MONP	Mono-oxo-isononyl phthalate	NA	Phthalate and Phthalate Alternative Metabolites
1412411-10-9	MPCHP	Mono-2-(propyl-6-carboxy-hexyl)-phthalate	NA	Phthalate and Phthalate Alternative Metabolites
1372605-11-2	MPHHP	Mono-2-(propyl-6-hydroxy-heptyl)-phthalate	NA	Phthalate and Phthalate Alternative Metabolites
1373125-92-8	MPOHP	Mono-2-(propyl-6-oxoheptyl)-phthalate	NA	Phthalate and Phthalate Alternative Metabolites
90-15-3	NAP1	1-Hydroxynaphthalene	NA	Polycyclic Aromatic Hydrocarbon (PAH) Metabolites
135-19-3	NAP2	2-Hydroxynaphthalene	NA	Polycyclic Aromatic Hydrocarbon (PAH) Metabolites
17708-87-1	NCOTT	Norcotinine (total)	NA	Tobacco Metabolites
190604-92-3	NDMA	N-Desmethyl-acetamiprid	Neonicotinoid insecticides	Pesticides and Metabolites
171103-04-1	NDMT	N-Desmethyl thiamethoxam	Neonicotinoid insecticides	Pesticides and Metabolites
54-11-5	NICT	Nicotine (total)	NA	Tobacco Metabolites
150824-47-8	NIT	Nitenpyram	Neonicotinoid insecticides	Pesticides and Metabolites
5746-86-1	NNICT	Nornicotine (total)	NA	Tobacco Metabolites
2820-55-5	NOXT	Nicotine 1’-Oxide (total)	NA	Tobacco Metabolites
115086-54-9	OFIMI	Imidacloprid-olefin	Neonicotinoid insecticides	Pesticides and Metabolites
155802-61-2	OHIMI	5-Hydroxyimidacloprid	Neonicotinoid insecticides	Pesticides and Metabolites
948-71-0	OHTBZ	5-Hydroxythiabendazole	Fungicides and metabolites	Pesticides and Metabolites
3739-38-6	PBA	3-Phenoxybenzoic acid	Pyrethroid metabolites	Pesticides and Metabolites
87-86-5	PCP	Pentachlorophenol	Fungicides and metabolites	Pesticides and Metabolites
2433-56-9	PHEN1	1-Hydroxyphenanthrene	NA	Polycyclic Aromatic Hydrocarbon (PAH) Metabolites
605-55-0	PHEN2	2-Hydroxyphenanthrene	NA	Polycyclic Aromatic Hydrocarbon (PAH) Metabolites
605-87-8	PHEN3	3-Hydroxyphenanthrene	NA	Polycyclic Aromatic Hydrocarbon (PAH) Metabolites
100-02-7	PNP	4-Nitrophenol	Organophosphorus insecticides: Specific metabolites	Pesticides and Metabolites
94-13-3	PRPB	Propyl paraben	Parabens	Personal Care and Consumer Product Chemicals and Metabolites

5315-79-7	PYR1	1-Hydroxypyrene	NA	Polycyclic Aromatic Hydrocarbon (PAH) Metabolites
4775-80-8	SPMA	N-Acetyl-S-phenyl-L-cysteine	NA	Volatile Organic Compound (VOC) Metabolites
101-20-2	TCC	Triclocarban	Antimicrobials	Personal Care and Consumer Product Chemicals and Metabolites
6515-38-4	TCP	3,5,6-Trichloro-2-pyridinol	Organophosphorus insecticides: Specific metabolites	Pesticides and Metabolites
3380-34-5	TCS	Triclosan	Antimicrobials	Personal Care and Consumer Product Chemicals and Metabolites
59042-50-1	TDCCA	Trans-3-(2,2-dichlorovinyl)-2,2-dimethyl-cyclopropane-1-carboxylic acid	Pyrethroid metabolites	Pesticides and Metabolites
1469-48-3	THPI	Cis-1,2,3,6-Tetrahydrophthalimide	Fungicides and metabolites	Pesticides and Metabolites
