# Supplementary material for: Early Pregnancy Targeted Exposome: Biological Response and Maternal BMI
Source: Toxics. 2026 May 12;14(5):421. doi: 10.3390/toxics14050421 (PMC13211517; doi:10.3390/toxics14050421)
Supplement: Supplementary file 1 [file toxics-14-00421-s001.zip › Supplementary Table S2 Lifestyle and BMI groups.pdf]

Supplementary Table S2: Lifestyle factors and BMI groups

		**Low BMI**		**High BMI**		**Total**	
		N	%	N	%	N	%
**Race**							
Black/African American		12	18.75%	9	16.36%	21	17.6%
Hispanic		8	12.5%	2	3.63%	10	8.4%
More Than One Race		0	0%	1	1.81%	1	0.8%
Non-Hispanic White		44	68.75%	43	78.1%	87	73.1%
**Smoker**	No	62	96.90%	50	90.90%	112	94.10%
	Yes	2	3.10%	4	7.30%	6	5.00%
	Missing	0	0.00%	1	1.80%	1	0.80%
**Smoke/day**	0/Day	62	96.90%	53	96.40%	115	96.60%
	1-3/Day	1	1.60%	0	0.00%	1	0.80%
	1/day	0	0.00%	1	1.80%	1	0.80%
	3/day	1	1.60%	0	0.00%	1	0.80%
	every 3 days	0	0.00%	1	1.80%	1	0.80%
**Tobacco Exposure in Last 3 Month**	No	56	87.50%	52	94.50%	108	90.80%
	Yes	8	12.50%	3	5.50%	11	9.20%
**Living Near Landfill**	No	61	95.30%	54	98.20%	115	96.60%
	Not sure	1	1.60%	0	0.00%	1	0.80%
	I don't think	0	0.00%	1	1.80%	1	0.80%
	Potentially	1	1.60%	0	0.00%	1	0.80%
	Yes	1	1.60%	0	0.00%	1	0.80%
**Use of Perfumes and Cosmetics**	No	14	21.90%	8	14.50%	22	18.50%
	Yes	48	75.00%	47	85.50%	95	79.80%
	Missing	2	3.10%	0	0.00%	2	1.70%
**Haircare Products**	No	19	29.70%	9	16.40%	28	23.50%
	Yes	43	67.20%	46	83.60%	89	74.80%
	Missing	2	3.10%	0	0.00%	2	1.70%
**Dental Fillings in last 3 Months**	No	61	95.30%	50	90.90%	111	93.30%
	Yes	3	4.70%	5	9.10%	8	6.70%
**Can Foods Consumption**	No	30	46.90%	23	41.80%	53	44.50%
	Yes	33	51.60%	32	58.20%	65	54.60%
	Missing	1	1.60%	0	0.00%	1	0.80%
**Can Food Frequency**	1 serving or less/day	33	51.60%	28	50.90%	61	51.30%
	1 serving or less/week	0	0.00%	1	1.80%	100.00%	0.80%
	2-3 serving/day	2	3.10%	2	3.60%	400.00%	3.40%
	No can food	29	45.30%	24	43.60%	5300.00%	44.50%
**Fast Food Consumption**	No	39	60.90%	28	50.90%	67	56.30%

	Yes	25	39.10%	26	47.30%	51	42.90%
	Missing	0	0.00%	1	1.80%	1	0.80%
**Fast Food Frequency**	<1/week	1	1.60%	0	0.00%	1	0.80%
	1/week	19	29.70%	20	36.40%	39	32.80%
	2-3/week	4	6.30%	5	9.10%	9	7.60%
	>4/week	1	1.60%	2	3.60%	3	2.50%
	No fast food	38	59.40%	28	50.90%	66	55.50%
	Missing	1	1.60%	0	0.00%	1	0.80%
**Fresh Vegetable Consumption**	No	1	1.60%	1	1.80%	2	1.70%
	Yes	63	98.40%	54	98.20%	117	98.30%
**Fresh Vegetable Frequency**	0 /day	1	1.60%	0	0.00%	1	0.80%
	1-3 ser/day	43	67.20%	43	78.20%	86	72.30%
	4-5 ser/day	16	25.00%	9	16.40%	25	21.00%
	At least once a week	1	1.60%	0	0.00%	1	0.80%
	Missing	3	4.70%	3	5.50%	6	5.00%
**Experience of Stress**	No	32	50.00%	26	47.30%	58	48.70%
	Yes	29	45.30%	24	43.60%	53	44.50%
	Missing	3	4.70%	5	9.10%	8	6.70%

Supplementary Table S2: Maternal Characteristics and Lifestyle Factors in Low and High BMI groups
