# Supplementary material for: Early Pregnancy Targeted Exposome: Biological Response and Maternal BMI
Source: Toxics. 2026 May 12;14(5):421. doi: 10.3390/toxics14050421 (PMC13211517; doi:10.3390/toxics14050421)
Supplement: Supplementary file 1 [file toxics-14-00421-s001.zip › Supplementary Table S4 t test Exposure Classes.pdf]

Supplementary Table S4: Differences in urinary levels -Exposure classes

	t.stat (H vs L)	p.val	FDR
**Volatile Organic Compounds**	-3.514	0.0006***	0.011
**Phytoestrogens**	-2.346	0.021*	0.100
**Parabens**	-2.296	0.023*	0.100
**Flame Retardants**	-1.980	0.050	0.122
**Phthalate and Alternatives**	-1.977	0.050	0.122
**Tobacco Smoke**	-1.668	0.098	0.188
**Organophosphorus Insecticides**	-1.635	0.105	0.188
**UV filters**	-1.605	0.111	0.188
**Neonicotinoid Insecticides**	-1.559	0.122	0.188
**Bisphenols**	-1.343	0.182	0.228
**Insect Repellents**	-1.330	0.186	0.228
**Fungicides**	-1.304	0.195	0.228
**Polycyclic Aromatic Hydrocarbons**	-1.251	0.214	0.228
**Pyrethroid Pesticides**	-1.248	0.215	0.228
**Antimicrobials**	-1.130	0.261	0.261
**Biological Response Markers**			
**Stress Markers**	-2.716	0.008**	0.065
**Oxidative Stress**	-2.086	0.039*	0.122

P< 0.05 = *, P<0.01 =**, P<0.001=***

Supplementary Table S4: Differences in urinary levels -Exposure classes.
