# Supplementary material for: Early Pregnancy Targeted Exposome: Biological Response and Maternal BMI
Source: Toxics. 2026 May 12;14(5):421. doi: 10.3390/toxics14050421 (PMC13211517; doi:10.3390/toxics14050421)
Supplement: Supplementary file 1 [file toxics-14-00421-s001.zip › Supplementary Table S5 Exposure Classes and BMI Associations.pdf]

Supplementary Table S5: Exposure classes and BMI associations

Exposure Class	BMI (Continuous)			BMI Groups		
**log transformed**	Corr Coeff	p-val	FDR	Corr Coeff	p-val	FDR
**Bisphenols**	-0.159	0.083	0.104	-0.123	0.183	0.241
**UV Filters**	-0.166	0.071	0.107	-0.146	0.112	0.166
**Flame Retardants**	-0.176	0.055	0.103	-.181*	0.049	0.084
**Antimicrobials**	-0.166	0.071	0.107	-0.104	0.261	0.261
**Parabens**	-.258**	0.005	0.038	-.207*	0.024	0.059
**Fungicides**	-0.143	0.122	0.141	-0.12	0.194	0.249
**Insect Repellents**	-0.14	0.129	0.141	-0.122	0.186	0.241
**Neonicotinoid Insecticides**	-.192*	0.037	0.111	-0.143	0.121	0.17
**Organophosphorus Insecticides**	-.236**	0.01	0.050	-0.149	0.106	0.161
**Pyrethroid Pesticides**	-.188*	0.041	0.088	-0.124	0.18	0.24
**Phthalate and alternatives**	-.210*	0.022	0.083	-0.179	0.051	0.085
**Phytoestrogens**	-.188*	0.041	0.088	-.212*	0.021	0.059
**Polycyclic Aromatic Hydrocarbons**	-0.111	0.23	0.230	-0.115	0.211	0.253
**Tobacco**	-0.171	0.063	0.105	-0.153	0.097	0.156
**Volatile Organic Compounds**	-.332**	<.001	0.015	-.310**	0.001	0.015

P< 0.05 = *, P<0.01 =**

Supplementary Table S5: Exposure classes and BMI associations.
