# Supplementary material for: Early Pregnancy Targeted Exposome: Biological Response and Maternal BMI
Source: Toxics. 2026 May 12;14(5):421. doi: 10.3390/toxics14050421 (PMC13211517; doi:10.3390/toxics14050421)
Supplement: Supplementary file 1 [file toxics-14-00421-s001.zip › Supplementary Table S6 cor and T test.pdf]

Supplementary Table S6: Differences in the individual exposure markers in low and high BMI groups.

Name	Corr coefficient	t-stat	p-val	FDR
**CYMA**	0.36126	4.1907	5.43E-05	0.005209
**HEMA2**	0.31298	3.5644	0.000529	0.025392
**PNP**	0.27687	3.1166	0.002302	0.035259
**MEHHP**	0.27371	3.0782	0.002595	0.035259
**HPMA2**	0.27287	3.068	0.002678	0.035259
**CEMA**	0.27124	3.0482	0.002847	0.035259
**MEOHP**	0.26839	3.0136	0.003165	0.035259
**MPOHP**	0.26772	3.0056	0.003244	0.035259
**PCP**	0.26721	2.9994	0.003306	0.035259
**AAMA**	0.2601	2.9137	0.004281	0.041093
**MECPP**	0.25444	2.8459	0.005231	0.045651
**NNICT**	0.25029	2.7963	0.006044	0.048355
**MNBP**	0.24648	2.751	0.006887	0.050857
**HPMA**	0.24236	2.7021	0.007916	0.054282
**DAZ**	0.23134	2.5722	0.011358	0.069717
**MMP**	0.23064	2.5638	0.01162	0.069717
**MIBP**	0.22847	2.5384	0.012449	0.070299
**PYR1**	0.22623	2.5122	0.013362	0.070952
**IMPY**	0.22304	2.4749	0.014762	0.070952
**BPS**	0.22299	2.4744	0.014782	0.070952
**DETP**	0.21964	2.4352	0.016393	0.072375
**MBZP**	0.21925	2.4307	0.016586	0.072375
**FLUO2**	0.21541	2.386	0.018636	0.075152
**MEOHTP**	0.21509	2.3823	0.018814	0.075152
**MECPTP**	0.21378	2.3671	0.019571	0.075152

Supplementary Table S6: Differences in the individual exposure markers in low and high BMI groups.
