# Supplementary material for: Early Pregnancy Targeted Exposome: Biological Response and Maternal BMI
Source: Toxics. 2026 May 12;14(5):421. doi: 10.3390/toxics14050421 (PMC13211517; doi:10.3390/toxics14050421)
Supplement: Supplementary file 1 [file toxics-14-00421-s001.zip › Supplementary Table S8 Exposures and Glucocorticoids VIP.pdf]

Supplementary Table S8: Exposure analytes and Glucocorticoid Stress markers VIP.

Top 25 Low BMI	Chemical class	VIP	Top 25 High BMI	Chemical class	VIP
**NAP2**	Polycyclic Aromatic Hydrocarbon Metabolites	1.693	**ETL**	Phytoestrogens and Metabolites	2.108
**FLUO2**	Polycyclic Aromatic Hydrocarbon Metabolites	1.567	**MMP**	Phthalate and Phthalate Alternative Metabolites	2.083
**PYR1**	Polycyclic Aromatic Hydrocarbon Metabolites	1.54	**HEMA2**	Volatile Organic Compound (VOC) Metabolites	2.016
**D24**	Herbicides and metabolites	1.53	**DHBMA**	Volatile Organic Compound (VOC) Metabolites	1.876
**AAMA**	Volatile Organic Compound (VOC) Metabolites	1.503	**AAMA**	Volatile Organic Compound (VOC) Metabolites	1.697
**MMP**	Phthalate and Phthalate Alternative Metabolites	1.457	**NNICT**	Tobacco Metabolites	1.68
**DPHP**	Flame Retardant Metabolites	1.447	**DEP**	Organophosphorus insecticides	1.677
**NNICT**	Tobacco Metabolites	1.438	**DETP**	Organophosphorus insecticides	1.593
**MEOHP**	Phthalate and Phthalate Alternative Metabolites	1.431	**CINA6**	Neonicotinoid insecticides	1.589
**COTT**	Tobacco Metabolites	1.399	**BCETP**	Flame Retardant Metabolites	1.58
**PHEN3**	Polycyclic Aromatic Hydrocarbon Metabolites	1.391	**NDMA**	Neonicotinoid insecticides	1.564
**MEHHP**	Phthalate and Phthalate Alternative Metabolites	1.388	**DMP**	Organophosphorus insecticides	1.492
**CINA6**	Neonicotinoid insecticides	1.38	**DMTP**	Organophosphorus insecticides	1.475

**MNBP**	Phthalate and Phthalate Alternative Metabolites	1.379	**PCP**	Fungicides and metabolites	1.47
**DBUP**	Flame Retardant Metabolites	1.374	**TCP**	Organophosphorus insecticides: Specific metabolites	1.424
**NCOTT**	Tobacco Metabolites	1.358	**MDA**	Organophosphorus insecticides: Specific metabolites	1.353
**MEP**	Phthalate and Phthalate Alternative Metabolites	1.354	**DMDP**	Organophosphorus insecticides: Specific metabolites	1.336
**PHEN2**	Polycyclic Aromatic Hydrocarbon Metabolites	1.35	**HPMA**	Volatile Organic Compound (VOC) Metabolites	1.312
**MECPP**	Phthalate and Phthalate Alternative Metabolites	1.333	**PHEN1**	Polycyclic Aromatic Hydrocarbon Metabolites	1.254
**MBZP**	Phthalate and Phthalate Alternative Metabolites	1.329	**NAP2**	Polycyclic Aromatic Hydrocarbon Metabolites	1.181
**HCOTT**	Tobacco Metabolites	1.325	**D24**	Herbicides and metabolites	1.174
**NICT**	Tobacco Metabolites	1.315	**PNP**	Organophosphorus insecticides: Specific metabolites	1.174
**HPMA**	Volatile Organic Compound (VOC) Metabolites	1.311	**DBUP**	Flame Retardant Metabolites	1.138
**MEHP**	Phthalate and Phthalate Alternative Metabolites	1.295	**DAZ**	Phytoestrogens and Metabolites	1.131
**MEPB**	Parabens	1.28	**PHEN2**	Polycyclic Aromatic Hydrocarbon Metabolites	1.125

Supplementary Table S8: Exposure analytes and Glucocorticoid Stress markers VIP.
