# Supplementary material for: Early Pregnancy Targeted Exposome: Biological Response and Maternal BMI
Source: Toxics. 2026 May 12;14(5):421. doi: 10.3390/toxics14050421 (PMC13211517; doi:10.3390/toxics14050421)
Supplement: Supplementary file 1 [file toxics-14-00421-s001.zip › Supplementary Table S9 Exposures and Oxidative Stress VIP.pdf]

*Supplementary Table S9: Exposure analytes and Oxidative Stress VIP*

| Top 25 Low BMI | Chemical class | VIP | Top 25 High BMI | Chemical class | VIP |
|---|---|---|---|---|---|
| **DPHP** | Flame Retardant Metabolites | 2.082 | **GNS** | Phytoestrogens and Metabolites | 2.42 |
| **CINA6** | Neonicotinoid insecticides | 1.958 | **DAZ** | Phytoestrogens and Metabolites | 2.309 |
| **DBUP** | Flame Retardant Metabolites | 1.852 | **OHTBZ** | Fungicides and metabolites | 1.937 |
| **PNP** | Organophosphorus insecticides | 1.801 | **PHEN2** | Polycyclic Aromatic Hydrocarbon Metabolites | 1.809 |
| **MMP** | Phthalate and Phthalate Alternative Metabolites | 1.654 | **AAMA** | Volatile Organic Compound (VOC) Metabolites | 1.759 |
| **AAMA** | Volatile Organic Compound (VOC) Metabolites | 1.644 | **PHEN3** | Polycyclic Aromatic Hydrocarbon Metabolites | 1.741 |
| **DEP** | Organophosphorus insecticides: Specific metabolites | 1.582 | **PHEN1** | Polycyclic Aromatic Hydrocarbon Metabolites | 1.647 |
| **MCINP** | Phthalate and Phthalate Alternative Metabolites | 1.556 | **DETP** | Organophosphorus insecticides | 1.631 |
| **HPMA** | Volatile Organic Compound (VOC) Metabolites | 1.539 | **HPMA** | Volatile Organic Compound (VOC) Metabolites | 1.601 |
| **PHEN3** | Polycyclic Aromatic Hydrocarbon Metabolites | 1.512 | **CINA6** | Neonicotinoid insecticides | 1.598 |
| **MPOHP** | Phthalate and Phthalate Alternative Metabolites | 1.486 | **NAP2** | Polycyclic Aromatic Hydrocarbon Metabolites | 1.596 |
| **TCP** | Organophosphorus insecticides | 1.475 | **DHBMA** | Volatile Organic Compound (VOC) Metabolites | 1.565 |
| **PHEN2** | Polycyclic Aromatic Hydrocarbon Metabolites | 1.474 | **NDMA** | Neonicotinoid insecticides | 1.497 |

| | | | | | |
|---|---|---|---|---|---|
| **D24** | Herbicides and metabolites | 1.464 | **DEP** | Organophosphorus insecticides | 1.472 |
| **DHBMA** | Volatile Organic Compound (VOC) Metabolites | 1.445 | **EQU** | Phytoestrogens and Metabolites | 1.462 |
| **MPHHP** | Phthalate and Phthalate Alternative Metabolites | 1.391 | **HEMA2** | Volatile Organic Compound (VOC) Metabolites | 1.428 |
| **MCPP** | Phthalate and Phthalate Alternative Metabolites | 1.391 | **ETL** | Phytoestrogens and Metabolites | 1.421 |
| **PHEN1** | Polycyclic Aromatic Hydrocarbon Metabolites | 1.377 | **ACE** | Neonicotinoid insecticides | 1.417 |
| **BPA** | Bisphenols | 1.368 | **TCP** | Organophosphorus insecticides | 1.383 |
| **HEMA2** | Volatile Organic Compound (VOC) Metabolites | 1.355 | **PNP** | Organophosphorus insecticides: Specific metabolites | 1.373 |
| **MECPTP** | Phthalate and Phthalate Alternative Metabolites | 1.279 | **NNICT** | Tobacco Metabolites | 1.352 |
| **MONCH** | Phthalate and Phthalate Alternative Metabolites | 1.27 | **DMP** | Organophosphorus insecticides | 1.333 |
| **PYR1** | Polycyclic Aromatic Hydrocarbon Metabolites | 1.258 | **MCPP** | Phthalate and Phthalate Alternative Metabolites | 1.314 |
| **MCOCH** | Phthalate and Phthalate Alternative Metabolites | 1.255 | **PYR1** | Polycyclic Aromatic Hydrocarbon Metabolites | 1.301 |
| **DETP** | Organophosphorus insecticides: Specific metabolites | 1.217 | **MPOHP** | Phthalate and Phthalate Alternative Metabolites | 1.268 |

Supplementary Table S9: Exposure analytes and Oxidative Stress VIP.
